# Weakly Circadian Cells Improve Resynchrony

**DOI:** 10.1371/journal.pcbi.1002787

**Published:** 2012-11-29

**Authors:** Alexis B. Webb, Stephanie R. Taylor, Kurt A. Thoroughman, Francis J. Doyle, Erik D. Herzog

**Affiliations:** 1 Department of Biology, Washington University, St. Louis, Missouri, United States of America; 2 Department of Computer Science, Colby College, Waterville, Maine, United States of America; 3 Department of Biomedical Engineering, Washington University, St. Louis, Missouri, United States of America; 4 Department of Chemical Engineering, University of California Santa Barbara, Santa Barbara, California, United States of America; Indiana University, United States of America

## Abstract

The mammalian suprachiasmatic nuclei (SCN) contain thousands of neurons capable of generating near 24-h rhythms. When isolated from their network, SCN neurons exhibit a range of oscillatory phenotypes: sustained or damping oscillations, or arrhythmic patterns. The implications of this variability are unknown. Experimentally, we found that cells within SCN explants recover from pharmacologically-induced desynchrony by re-establishing rhythmicity and synchrony in waves, independent of their intrinsic circadian period We therefore hypothesized that a cell's location within the network may also critically determine its resynchronization. To test this, we employed a deterministic, mechanistic model of circadian oscillators where we could independently control cell-intrinsic and network-connectivity parameters. We found that small changes in key parameters produced the full range of oscillatory phenotypes seen in biological cells, including similar distributions of period, amplitude and ability to cycle. The model also predicted that weaker oscillators could adjust their phase more readily than stronger oscillators. Using these model cells we explored potential biological consequences of their number and placement within the network. We found that the population synchronized to a higher degree when weak oscillators were at highly connected nodes within the network. A mathematically independent phase-amplitude model reproduced these findings. Thus, small differences in cell-intrinsic parameters contribute to large changes in the oscillatory ability of a cell, but the location of weak oscillators within the network also critically shapes the degree of synchronization for the population.

## Author Summary

Circadian rhythms are daily, near 24-h oscillations in biological processes that nearly all organisms on Earth experience. Single cells contain a molecular clock that drives circadian rhythms in physiology and, when many cells synchronize in a population, daily behaviors. We hypothesized that small differences in intrinsic cellular properties allow for a diversity of circadian periods and amplitudes across cells. We observed circadian cells and their synchrony before, during, and after limiting communication between cells and then compared their intrinsic properties to their resynchronization behavior. We found that arrhythmic, weakly oscillating, and self-sustained circadian cells rejoined the rhythmic population independent of their cell-intrinsic oscillations. Using a mechanistic computational model of circadian cells, we found that resynchronization could be enhanced by including more weak oscillators or by placing weak oscillators at more connected nodes in the network. We conclude that intrinsic properties (e.g. oscillator weakness and responsiveness) and network structure (e.g. positions of weak oscillators) can independently buffer tissue rhythms from perturbations. This reveals how cellular and network properties impose rules on systems of circadian cells that must achieve synchrony from a desynchronized state, for example during perinatal development or when forced to overcome societal constraints on sleep-wake behavior, such as working early or late shifts.

## Introduction

Circadian clocks generate the near 24-h oscillations that orchestrate daily behaviors in organisms throughout the kingdoms of life [1]. In mammals, the suprachiasmatic nucleus (SCN), a bilateral structure of 20,000 neurons in the ventral hypothalamus, functions as the master pacemaker with circadian cells driving rhythms in behavior and physiological processes, such as sleep-wake, locomotor activity, temperature, and hormone release [2]. It was hypothesized that every SCN neuron acts an autonomous clock, using molecular feedback loops to generate daily rhythms in gene expression and cellular output in the absence of external signals [3], [4], [5], [6], [7]. For example, the *Period2* (*Per2*) gene, a clock gene found in humans and other animals, shows daily rhythms in transcription that appear to depend on daily repression by complexes including its protein (PER2) [8], [9]. Recent data, however, highlight that, when isolated, SCN neurons exhibit a range of behaviors including damped or unstable circadian oscillations [10], [11]. Therefore, although all cells may be capable of autonomous rhythmicity, they require stabilization from the SCN network to function as robust circadian oscillators. The potential source or sources of this cell-intrinsic variability, as well as its potential impact, are unknown. Whether the intrinsic properties of SCN oscillators independent of, or interactions amongst groups of oscillators within, the SCN network, or both, are responsible for the overall behavior is a current area of research [12], [13].

To first test the hypothesis that intrinsic differences between cells may affect how they resynchronize to each other, we followed daily oscillations of PERIOD2 protein levels in single SCN cells before, during, and after pharmacological blockade of intercellular signaling. The results revealed individual cells that differed in their intrinsic amplitude, level of gene expression, circadian period and ability to sustain rhythmicity, none of which predicted the cells' behaviors as they resynchronized to the population. Instead, we found that oscillations resumed and cells joined the rhythmic population at specific circadian phases, ultimately revealing the previously described daily waves of gene expression across the SCN [6], [14], [15]. Recent work has further suggested that the phase relationships of SCN cells across the network could be important for robust rhythmic behavior of the tissue [13], [16], [17].

To understand the complex behaviors of SCN cells, many studies have employed computational models. Both deterministic models detailing the molecular processes driving oscillations in single cells and stochastic models investigating the effects of noise on the system have aided in the understanding of mechanisms generating circadian rhythmicity in mammals [18], [19], [20]. Multi-cellular network models have been constructed from these single oscillators to describe synchrony across the SCN tissue, entrainment to light-dark cycles, and phase shifting behavior [21], [22], [23], [24]. Network models have also probed regional differences in the SCN [25], [26] and the phenomenon of splitting, in which synchronized regions in the SCN can oscillate with the same period but opposite phases [27]. We were interested in the relationship between cell-intrinsic rhythmicity and tissue synchronization, and found two major implications in the literature. First is that “smaller is better”: damped oscillators [23], [24], [28] and oscillators with short relaxation times [29] synchronize efficiently. Additionally, a recent study of fibroblast cells shows that cellular oscillators have small, but sustained amplitudes, and that their proximity to a bifurcation allows them greater control over their period [30]. The authors note that this could be advantageous for peripheral oscillators that need to be entrained by the pacemaker and suggest that similar properties in pacemaker cells could aid synchrony. Second, network topology also affects the quality of synchrony, and specifically, small-world type network topologies are beneficial for synchrony [22], [26]. It has not been shown, however, why small oscillations are good for synchrony or how cell-intrinsic behaviors and network topology together affect synchrony. Using a mathematical model provides us the flexibility to explain biological phenomena without constraints found in the physiology, e.g. the type, number, and location of oscillators within a network. We sought to address this by first assessing the roles of intracellular processes on intrinsic properties, such as rhythmic ability and phase-responsiveness. Next we assessed the effects of individual cell properties on network synchronization, and finally, how the location of key cells within the network affects synchrony.

We hypothesized that intracellular properties and intercellular interactions contribute to the resynchronization behaviors we observed in the tissue data. To test this prediction, we used a computational model to simulate clock gene transcription-translation feedback loops in single cells and found that small changes in parameter combinations produce the range of intrinsic oscillations observed in SCN cells. When placed in a network, these cells were able to synchronize, meaning that they were capable of adjusting their phases to align with the population. To understand this phenomenon, we computed velocity response curves (VRCs) for these cells [31], [32]. VRCs predict the phase velocity, i.e. how fast phase changes in response to intercellular signals. For our model, the VRCs suggested that cells with weaker oscillations could adjust their phase velocity more readily than cells with strong oscillations. These results were consistent with previous results that “smaller” is better to initiate synchrony, but with an alternative definition of smaller – we studied the effects of rhythmic, but low-amplitude (weak) cells, rather than initially rhythmic cells that lose amplitude, and eventually, all rhythmic ability, over the long-term (damped). We therefore tested the prediction that inclusion of weak circadian cells, which are highly responsive when isolated, would improve a network's ability to synchronize. We hypothesized that as weak cells establish rhythmicity and synchrony in the network, they lose responsiveness, becoming strong oscillators when coupled. By using a model of 400 coupled, heterogeneously oscillating cells, we found that increasing the proportion of weak oscillators or placing weak oscillators at more connected nodes in the network allowed for improved resynchronization.

## Results

### Circadian cells desynchronize similarly, but resynchronize differently

**Figure 1:**
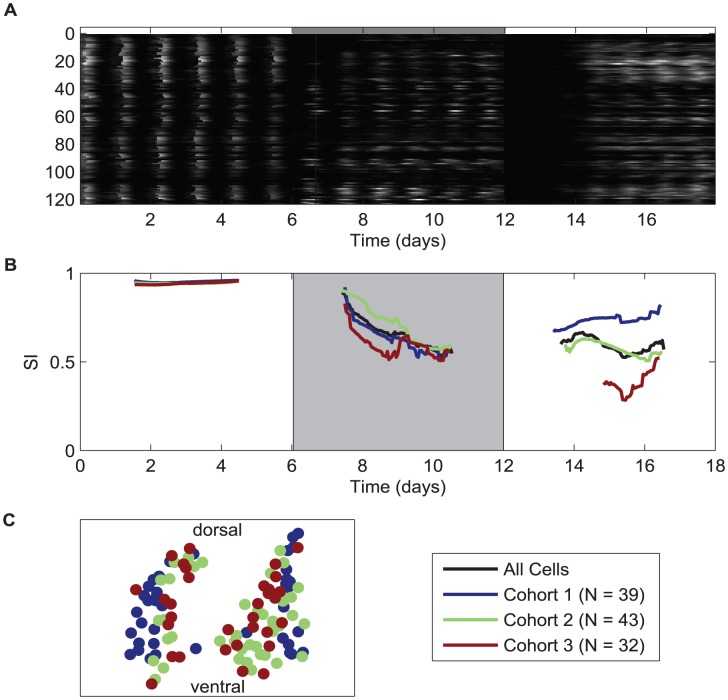
TTX-treatment and wash-out in SCN explants reveals differences in the dynamics of desynchrony and resynchrony of single cells. (**A**) Raster plot of bioluminescence (a. u.) from single neurons in an organotypic SCN explant before (days 1–6), during (days 7–12), and following treatment with 0.5 µM tetrodotoxin (TTX, days 13–18). Time of TTX treatment (days 7–12) is indicated by the shaded region of the bar along the top of the raster plot. TTX minimizes cell-cell communication and reveals intrinsic circadian properties. Here we plot traces from 123 neurons that show a range of oscillatory behaviors in TTX, all of which are rhythmic when coupling is in place before treatment. Following washout, rhythms return to all cells, but at different rates. The intensity of the white is proportional to the bioluminescence in each experimental condition. (**B**) Preliminary data suggests a small population of neurons resynchronizes first and then a slow recruitment of the remaining population is observed following TTX washout. Here we include the SI for the entire population (black), as well as the SI for each cohort of cells. Cohorts are determined by when cells become rhythmic after the wash; cells in cohort 1 (blue) become rhythmic first (hours 31–35), cells in cohort 2 (green) become rhythmic second (hours 36–45), and cells in cohort 3 (red) become rhythmic after hour 46 of the wash. (**C**) The location of single cells recorded in the SCN explant. Cell position is color-coded based on the resynchronization cohort.

Recent reports have shown that when SCN explants are treated with tetrodotoxin (TTX), a blocker of voltage-gated Na+ channels, the circadian rhythms of single cells gradually drift out of phase from one another [6], [10], [33], [34]. To understand the relative contributions of cell-intrinsic and network properties to these synchronization dynamics, we examined the bioluminescence recorded from single cells (n = 123 across two nuclei, slice 1; n = 90 within one nucleus, slice 2; for details see Text S1) in SCN explants from homozygous PERIOD2::LUCIFERASE (PER2::LUC) knock-in mice [35] during and after TTX treatment (Fig. 1). Although all cells appeared to gradually drift out of phase, only some expressed sustained circadian rhythms while others slowly or rapidly lost rhythmicity until the TTX was removed, at which point they began to regain rhythmicity and, eventually, synchrony. Looking at the timing of recovery of oscillations in slice 1, we found that approximately one-third of the cells that regained rhythms showed significant circadian oscillations within the first 35 h after removal of TTX. During the next 10 h another group of cells, similar in number, became circadian and began to synchronize to the first group. The remaining cells showed significant circadian rhythms starting around 45 h after removal of TTX, with the final cells entering by 96 h. Interestingly, the initial cohort of cells regained rhythmicity closely in phase while later cells regained rhythmicity with more broadly dispersed phases (Figs. 1B; Text S1; Rayleigh tests performed at the entrance time of the last cell in each cohort; Cohort 1, n = 39 cells, r = 0.68; Cohort 2, n = 43 cells, r = 0.55; Cohort 3, n = 32 cells, r = 0.43). In the second explant, we found a similar gradual restoration of rhythmicity to individual cells after TTX was removed (slice 2; Text S1). There was also a spatial pattern in each nucleus of the slices: lateral cells regain rhythmicity earlier than or phase lead medial cells (see Table S2 and Figure S10 in Text S1). In addition, lateral cells are, on average, smaller in amplitude than medial cells. This suggested a spatial organization of amplitude in the network during synchrony recovery. This led us to ask if there was something intrinsically different about the oscillations in cells that became rhythmic earlier or later after TTX was removed.

We reported previously that SCN cells uncoupled by TTX display diverse circadian behaviors both in terms of amplitude and period [10]. We acknowledge the possibility that TTX treatment itself can alter a cell's amplitude; however, we will assume that amplitude during TTX is reflective of amplitude that is independent of other feedback from other cells, and as such, is intrinsic to a cell. To determine whether or not intrinsic behaviors explain early or late restoration of rhythms, we compared amplitudes and periods of cells in TTX-treated SCN explants to the time when their rhythms reemerged and to the quality of synchrony within the group of circadian cells. In both slices, we found no significant correlations (R^2^ values of <0.2, Text S1) between intrinsic circadian properties, such as mean bioluminescence, total bioluminescence, bioluminescence amplitude and period, and when a cell joined in oscillations within a resynchronizing SCN network. We conclude that intrinsic properties alone fail to explain the dynamic emergence of rhythms and resynchrony of individual cells. Therefore network properties likely participate along with these intrinsic behaviors in synchrony. To explore the relationship, if any, between the intrinsic properties of the cells within the context of the network, we implemented a mathematical model.

### Small changes in key parameters can create diverse oscillator types

First, we sought to reproduce the diversity of characteristics of isolated cells (i.e. PER-driven bioluminescence with patterns that could be described as strongly rhythmic, weakly rhythmic or arrhythmic over multiple days) by identifying potential molecular determinants of these circadian phenotypes. We utilized an existing model of the mammalian molecular clock to simulate SCN neurons [18] and focused on four parameters that regulate the output we recorded in the biological data (PER2::LUC): the rate of transcription of the *Period* (*Per*) gene, or translation, phosphorylation or degradation of the PERIOD (PER) protein. We categorized each cell as arrhythmic, weak (rhythmic but low in amplitude), or strong (rhythmic and high in amplitude; see Materials and Methods). We found that changing any of the four parameters by at least 10% moved simulated cells from arrhythmic to weakly rhythmic to sustained circadian gene expression (Fig. S2A). Regardless of whether they were varied alone or in combination, these parameters recapitulated the phenotypes found in SCN explants (Figs. S1, S2, S3).

We used a multi-dimensional visualization technique to evaluate the relative contributions of the four parameters to rhythm generation [36], providing a novel analysis of sensitivity of strength and sustainability of circadian oscillations to specific parameter combinations. By nesting parameter combinations into stacks, we arranged our data set with a large number of parameters in two dimensions that could be displayed easily (Materials and Methods). Based on the position of cells across the parameter space visualization, we found that small changes in rates of transcription of *Per* mRNA and degradation of PER protein produced larger effects than changes in translation and phosphorylation of PER on the circadian phenotype of simulated cells (Figs. S1 and S2B). *Per* rhythmicity was similarly more sensitive to *Bmal1* transcription and BMAL1 degradation than BMAL1 translation and phosphorylation (Fig. S2B).

We ensured that individual model cells accurately represented individual cells from the slice. Amplitude was of particular importance because here we tested the effect of weak oscillators for the first time. When we compared the circadian periods and amplitudes of simulated and recorded cells we found no correlation between period and amplitude for either the model or the slices (Fig. S4; R^2^<0.02 for all). Further, the periods were similarly distributed (slice 1 std. dev. = 2.1 h, slice 2 std. dev. = 2.1 h, model std. dev. = 2.1 h) and the amplitude distributions were dominated by small values in both the model and the slices (Fig. S4, Text S1). This suggests that the period and amplitude values in model cells faithfully mimic behaviors we observe in the slice during TTX treatment. Neither this independence of period and amplitude, nor the dominance of small amplitudes has been described in other computational models. Here we are explicit in our modeling that the intrinsic amplitude is much smaller than the in-network amplitude. We concluded that by specifying small differences in key circadian parameters between cells, our simulated cells accurately represented the diverse rhythmic abilities, as well as realistic intrinsic properties such as period and amplitude, of SCN cells.

### Weak oscillators predicted to show greater shifts following perturbation

**Figure 2:**
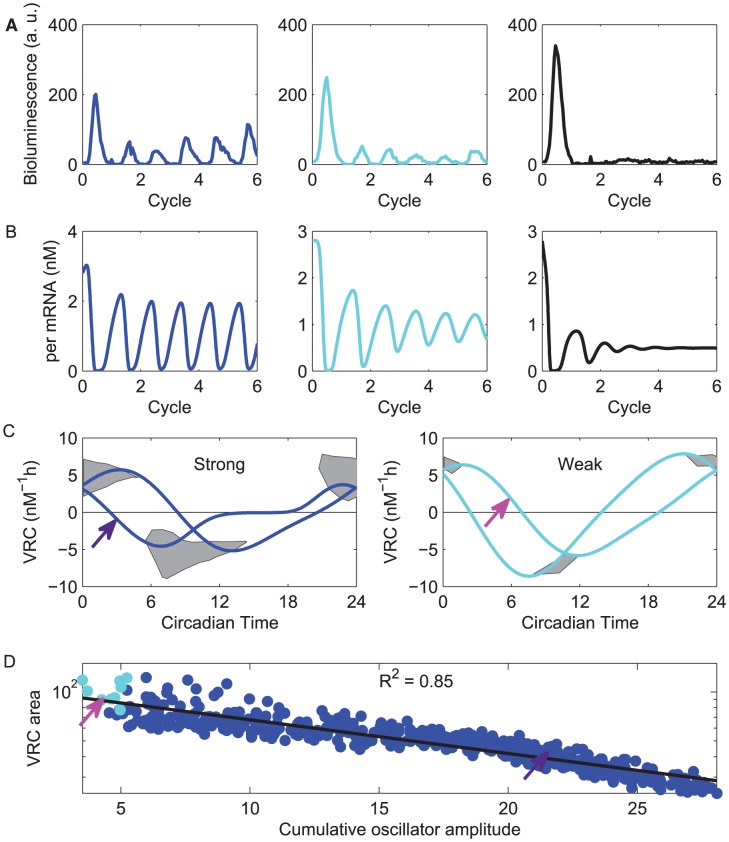
Mechanistic model produces diverse cells with varying response properties similar to SCN cells treated with TTX. (**A**) Bioluminescence traces from cells classified as strong (left), weak (center) or arrhythmic (right) based on their amplitude in their final cycle during TTX treatment. We show the last peak before TTX treatment and then 5 cycles during treatment. (**B**) Simulated traces using a mechanistic model from strong, weak, and arrhythmic cells show similar oscillation qualities to the real cells. Simulated cells are classified according to their limit cycle's “cumulative amplitude”, which is the sum of peak-to-trough amplitudes of all modeled messages and proteins. (**C**) The range of amplitudes and phases of velocity response curves (VRCs) from strong (left) and weak (right) simulated cells; VRCs are not computable for arrhythmic simulated cells. The shaded regions indicate the areas in which model VRCs peak and trough. Two representative VRC's are shown in each plot, each with a trough and peak falling in a different place in the regions. Circadian time is defined relative to peak *Per* mRNA expression, which is at circadian time (CT) 7. Arrows indicate the VRC's for the cells shown in (**B**). By computing the area under the absolute value of the VRC, we determine each cell's ability to speed up or slow down when signaled (greater area indicates greater “shiftability”). (**D**) We plot the VRC area (log scale) vs. the cumulative amplitude (linear scale). Arrows indicate the data points for the example cells in (**B**). We include the correlation coefficient for the amplitude and natural logarithm of the VRC area. There is a negative correlation between the VRC area and the oscillatory behavior of simulated cells, indicating that weak cells are more shiftable.

Another relevant property of a circadian oscillator is how it will adjust its phase velocity (speed) following a perturbation. We calculated the velocity response properties of the simulated cell set, including both strong and weak cells. Fig. 2C shows representative velocity response curves (VRCs) to a signal, where curves are plotted as a function of phase of oscillation. From the curve, we see that if the signal arrives early in the day (around circadian time, CT, 0) the cell will speed up, and if it arrives late in the day (between CT6 and CT12), the cell will slow down. To measure the cell's ability to shift, we computed the area under the absolute value of the VRC. We compared this VRC area to intrinsic oscillator amplitude (the sum of the peak to trough amplitude of all states) and found it inversely correlated with velocity response (Fig. 2D; R^2^ = 0.85). This indicates that oscillators with small intrinsic amplitude are more likely to have larger velocity response and therefore greater phase shifting ability compared to high-amplitude cells. Interestingly, we found that small oscillators in both the simulation and slice 1 have a broader distribution of periods compared to strong cells. The VRC results suggest a functional strategy to overcome this period variability: weaker cells are better at shifting their phase.

### Networks including weak oscillators in greater proportions or at more highly connected nodes reach higher synchrony

**Figure 3:**
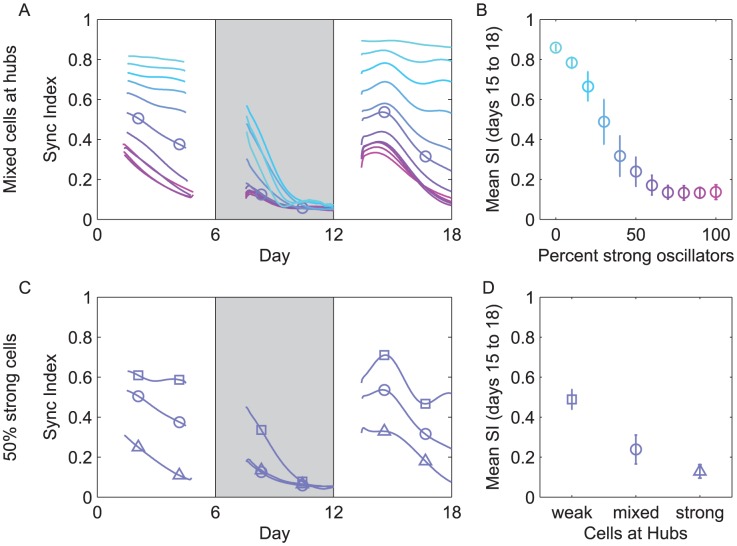
Networks with more weak oscillators resynchronize better. We measured the synchronization index (SI) of the modeled population in the coupled (days 1–6), TTX-uncoupled (days 7–12), and recovery (days 13–18) conditions and compare the results across different population and network configurations. We show the effects of varying the percentage of strong oscillators (top row) and of varying the network topology (bottom row). For each configuration-type, we run 56 simulations. (**A**) The mean SI is shown over time for populations of 0% (cyan), 20%, 35%, 50%, 65%, 80%, and 100% (magenta) strong cells. (**B**) For each simulation, we compute the mean SI for days 3 to 5 after the wash (days 15 to 18). Here we show the mean and standard deviation across simulations using the same color scheme. (**C**) We simulated all networks with 50% strong cells and show the mean SI is shown for populations with weak cells at hubs (squares), both strong and weak cells placed randomly at hubs (circles), and strong cells at hubs (triangles) The effect of network configuration on quality of synchrony is shown in (**D**) and is labeled by the type of cell at network hubs.

To test empirically if and how the proportion of weak oscillators contribute to the synchronization properties of a network like the SCN, we modeled a network of 400 SCN cells with diverse oscillatory abilities, including different periods and amplitudes, as well as network connections. Specifically, each cell had a unique set of parameters selected randomly to establish a population with defined proportions of arrhythmic, weak and sustained oscillators. We chose to include both local and global coupling between cells based on recent theoretical work [22]. Each cell was connected to its four nearest neighbors and 20% of cells connected to cells beyond their immediate neighbors (Fig. S5). Coupling was achieved in the model by simulating release of vasoactive intestinal polypeptide (VIP), a known synchronizer in the SCN [37], from all cells. Each network (n = 56 independent runs for each condition) was populated with 400 characterized cells and its overall response to uncoupling and recoupling was measured by calculating the synchronization index (SI) of all cells (see Materials and Methods). On average, we found that networks with more weak oscillators (total oscillator amplitude < = 8.4 a.u.) reliably reached higher levels of synchrony (SI> = 0.7 at days 15–18) with approximately 5-fold higher synchrony in networks comprised of mostly weak, compared to mostly strong, oscillators (Fig. 3A–B; ANOVA between populations, p<0.001). We found that networks with only strong oscillators failed to resynchronize (Fig. 3A–B; SI = 0.2 at days 15–18). We conclude that weak, highly shiftable cells can promote synchrony.

To test the importance of location within the network, we evaluated synchrony in networks of 50% weak and 50% sustained oscillators in which weak oscillators were assigned to hubs, i.e. nodes with more than the average of 10 outputs (range = 4–39 outputs). We found that when weak cells were placed in the more connected nodes of the network (n = 56 independent network runs for each condition), the population reached approximately 5-fold greater synchrony compared to networks with strong cells at these nodes or networks with oscillators distributed to nodes randomly (Fig. 3C–D ANOVA between populations, p<0.001). The quality of resynchrony, therefore, depended on both the number and placement of weak, shiftable oscillators in the network.

### Weak, low-amplitude oscillators enhance network synchrony similarly to damped oscillators

To test whether our findings regarding weak oscillators extend to damped oscillators, we repeated the simulations using cells that lose amplitude, and eventually, all rhythmic ability (see Materials and Methods). We found that the effects of weak circadian cells and of damped cells were nearly identical. For example, increasing the percentage of damped cells (Fig. S6A) or placing damped cells at network hubs (Fig. S6B) enhanced synchrony. Further, we verified that our results were not sensitive to our definition of weak. For the simulations used to generate Fig. 3 and Fig. S6, the weak cells were the smallest 30% in intrinsic amplitude. We repeated all simulations varying the percentage of rhythmic cells classified as weak. For each of these cut-offs, we measured the largest difference in SI between weak and damped cells (range = +0.07–0.69). The closer the weakly circadian cells were to the bifurcation, the more they acted like damped cells. We observed that as long as the cut-off is less than 50%, weak cells are similar to damped cells. To demonstrate that these behaviors could be generalized to other oscillatory systems, we constructed a phase-amplitude model, which functions as a reduced version of our mechanistic model (Text S2). We wanted to know if the benefit of weak cells for synchrony was evident in simpler systems and if a reduced model could further our understanding. The reduced model also showed that inclusion of more low amplitude, or small, oscillators or strategically placing them at more highly connected nodes increased synchrony. Thus, these results were robust across model compositions and types, and indicate that larger phase adjustments by small oscillators will, in general, produce higher synchrony.

## Discussion

### Potential sources and consequences of oscillator heterogeneity in a circadian network

Although physiologists and anatomists have described differences between SCN cells including their circadian amplitude, phase and waveform [38], [39], [40], the functional role of oscillator heterogeneity has been little studied. For example, the intrinsic daily oscillations of SCN neurons can be sustained, damped, or absent [10], [11], [41], but the consequences of these diverse circadian phenotypes remain unknown. Here, we found that the resynchronization of SCN cells following pharmacological blockade of cell-cell signaling involves waves of cells becoming rhythmic and adjusting their phases to join the daily cycling of the population. Previous theoretical studies have suggested that damped cells can aid network synchrony by entraining to a wider range of periods [23], [24] and relaxation oscillators can entrain faster if they have shorter relaxation rates or more spike-like waveforms [29], but have also highlighted that it is not yet possible to distinguish whether SCN cells should be modeled as damped oscillators or low amplitude, sustained oscillators [28]. What are the potential sources of these differences? By tuning a computational model, we found that small changes in a small set of parameters could produce a realistic distribution of cells that varied not only in period length, as has been generated previously [18], [21], but in qualities of the oscillations themselves. Using non-biased minimization techniques to represent multi-dimensional parameter space, we found that parameters associated with transcription rate and protein degradation of the *Period* gene were more likely to contribute to circadian changes than other parameters. We speculate that genetic differences in and the environmental modulation of these key rate constants between SCN cells could underlie the heterogeneity in their circadian properties. For example, it has been shown that the amplitude of *Per* transcription is altered in the absence of VIP [42] and that the stability of PER protein against degradation affects circadian period [43].

Because we found that the amplitude of our model cells is reliably and inversely related to their ability to adjust their phase velocities in response to natural signals, we tested the impact of both low (weak) and high (strong) amplitude oscillators on network synchrony. Previously, Bernard and colleagues suggested that a network comprised of damped circadian oscillators is capable of synchronizing and maintaining rhythmicity, and hypothesized that damped oscillators, when synchronized, induced rhythmicity in the population [24]. Locke and colleagues then performed a parameter optimization, searching to maximize the ability of a network of oscillators to synchronize. The best-synchronized networks were composed of damped cells [44]. Together, these results suggested that the driving force coupling cells together could arise from some inherent property found in damped cells.

In a similar fashion, we sought to identify inherent characteristics in both biological and modeled weak oscillators, including relationships between intrinsic amplitude and intrinsic shiftability. Published models using damped oscillators have been unable to mathematically quantify shiftability. By studying weak circadian oscillators, we measured larger changes in oscillator speed for smaller amplitude cells. Measurements of shiftability now provide a tool to study for the first time the kinetics of resynchronization. We posit that weakly oscillatory cells can send signals to other weakly oscillatory cells to readily adjust their phases. As the system synchronizes, the cells gain amplitude and thus lose the ability to make dramatic shifts. This suggests a strategy for neurons to resynchronize. The system can move from being sensitive to perturbations to being robust against them through a process of cell-cell amplification of rhythm amplitudes [33].

In our model networks we demonstrated that the total number of a specific oscillator type is critical and that there is an effect of the degree of connectivity of certain oscillator types on synchrony, such that, networks with more and more highly connected weak oscillators have improved synchrony during the recovery period following a perturbation. We concluded that heterogeneity arises from both cell intrinsic and network contributions, including the network topology and number of weakly circadian cells. The model does not account for all dynamics of resynchrony that we observed in the data, which will be addressed in the future. For example, though we observed populations of cells consistently ahead of or behind the mean phase of the network simulations, we observed no spatial pattern in these phase differences; the more homogeneous connections in the model networks led to most cells becoming rhythmic at the same time and together tighten in phase. Future work will use modeling to understand if and how spatial heterogeneity in network connections causes spatial patterns in the phase of oscillators across the slice. Future work will also take into account stochasticity in cell behavior. Preliminary results (data not shown) indicate that incorporating white noise into tissue simulations has no effect on the role of weak oscillators.

### Is the tissue the issue?

How the evolving differences within oscillators and amongst oscillator populations carry over to the behavior of networks is an open question for investigation. We return to the issue of whether rhythmicity and synchrony are due to intrinsic cell properties or are dependent on cell location and network structure. Recent studies have examined phase heterogeneity within the SCN [12], [13] and have concluded it is not a function of cellular properties. Foley and colleagues summarized their findings as “the tissue is the issue” – that placement within the SCN network (based on assigned phase) dictates whether and how an SCN neuron will oscillate [13]. We extend this to hypothesize specifically that cells, which are intrinsically different in their ability to maintain strong or weak rhythms, will impact the population rhythm differentially (e.g. the quality of synchronization increases with more weak oscillators), but also depending on their location within the network (e.g. cells at hubs have greater influence). Other theoretical studies have emphasized that the number of connections between cells could modulate the degree of synchrony in the network and argued for region-specific placement of particular oscillator types (e.g. sustained cells in the dorsal SCN and arrhythmic or gated cells in the ventral SCN) [25], [45], [46]. We find no evidence for specialized, localized populations of oscillators in the resynchronizing SCN slice following the removal of TTX. In contrast, our model shows the importance of weakly rhythmic, highly responsive oscillators at hubs where they can send coordinated phase information broadly throughout the network, becoming less responsive as they increase in amplitude, and that this is critical for improved synchrony. It is thought that SCN neurons establish rhythmicity and synchrony amongst each other and with the external light-dark environment late in gestation [47], [48]. Because these features are likely critical for the survival [49], we posit that the composition of the SCN, including a continuum of oscillator behaviors and connections, allows the tissue to adjust to shifts in environmental timing cues. These properties may be universal to all networks that include weak oscillators.

## Materials and Methods

### SCN cell culture

Single cells measured in SCN slices reported in this study were recorded as previously published [10]. Briefly, SCN explants from neonatal PER2::LUC mice were cultured for 3 days on MilliCell-CM (Millipore) membrane pieces in CO_2_-buffered medium supplemented with 10% newborn calf serum (Invitrogen) before being inverted onto polylysine/laminin coated coverslip dishes. All procedures were approved by the Washington University Animal Studies Committee and complied with NIH guidelines.

### Bioluminescence recording

We conducted recordings in air-buffered medium containing 0.1 mM beetle luciferin (BioThema) at 37°C beginning at day 2 after slice transfer to coverslip dishes We temporally (1 h integration time) and spatially (2×2 pixel resolution) bioluminescence counts using a Versarray 1024 CCD camera (Princeton Instruments).

### TTX treatment and washout

Following 6 days of baseline recording, we treated organotypic SCN explants with 0.5 µM tetrodotoxin (TTX, Sigma) as previously described [10]. TTX remained in the medium for 6 days before the medium was removed and we washed explants with 1 full volume exchange of fresh medium. Recording then continued for at least 6 days to examine rhythms as cells resynchronized after the restoration of cell-cell communication.

### Analysis of rhythms

We used NIH ImageJ software to process all images by first subtracting background levels and then measuring pixel intensity over time in a region of interest above each cell. Cells were tracked manually from frame-to-frame and across treatments to account for any tissue movement. Cells were initially scored as rhythmic or arrhythmic if their gene expression rhythm oscillated with a period between 15 and 35 hours that was statistically significant by both Chi-squared periodogram [50] and FFT-NLLS [51]. We also used Wavos to determine period and phase information from the single cell traces [52].

### Mechanistic model of molecular clock in single SCN cells

A version of a previously published 16-ordinary differential equation model of the mammalian circadian clock was used to simulate rhythms in single model cells [18]. We altered parameters for rates of transcription, translation, phosphorylation, and degradation of either *Period* or *Bmal1* genes, leaving 50 other parameters set to published basal values [18], and measured rhythms in gene output. We simulated 720 hours of gene expression from each cell, using initial conditions from a representative, high-amplitude sustained cell.

### Visualization of parameter space

To measure the sensitivity of circadian cycling to clock gene parameters, we organized results from single cell simulations using clutter based dimensional reordering (CBDR), which applies minimization and dimensional stacking algorithms described below. These methods allow visualization of the underlying structure of clock parameter space and gauge the influence of tested parameters relative to output behavior. We utilized published Matlab code [36] to minimize differences between output scores (strong, weak, arrhythmic) and cluster behaviors together. The code arranged parameter combinations iteratively until the minimization requirement, i.e. cells with like behavior, were clustered together, was fulfilled. First, the code scans one pair of parameters over a range of values while the remaining parameters are set to basal values. Then we label this grid based on the output for each combination and add it to a larger montage of other parameter pairs. For a useful visualization, the code orders these dimensional stacks to group similar outputs together. Given a unique behavior and parameter combination for each cell, we minimize the stack so that differences between regions of varying outputs are small (in this case, strong, weak, or arrhythmic patterns in gene expression), and this provides an order ranking of “higher” versus “lower” parameters in the stack. Changes in parameter value that produce larger effects in output phenotype are higher in the stack order.

### Velocity response curves

A velocity response curve (VRC) predicts the effect of parametric perturbation on the phase velocity of the oscillator. For a cell in the SCN, there is a single parameter (vsP) that is manipulated by VIP signaling. Hence, we consider the VRC associated with vsP, mapping the circadian time of VIP signaling to its effect on the phase velocity. Cells with higher-magnitude VRCs can be sped up or slowed down more by VIP signals than cells with lower magnitude VRCs. To quantify the “shiftability” of a cell, we compute the area under the absolute value of the VRC.

A VRC may be computed for any cell with a parameter set allowing for limit cycle oscillations. For details regarding computation, see [31].

### Categorization of simulated cell types

Mathematically, the individual cells we have modeled can be categorized as rhythmic (those that converge to a periodic orbit) or arrhythmic (those that converge to a steady-state solution). However, simulations of single cells display a spectrum of behaviors, with some showing lower or higher amplitude than others. Using the peak to trough amplitudes of all model components (i.e. by summing the amplitudes of all states), we separated the rhythmic cells into two categories: 1.Weak cells are rhythmic with small amplitudes and 2. Strong cells are rhythmic with larger amplitudes.

For some simulations, we needed damped oscillators, which form a subset of the arrhythmic oscillators. We used the total amplitude at the end of a 15-day simulation (starting from high-amplitude initial conditions) to create the additional categories: 1. Flat cells are arrhythmic and have the smallest amplitudes in their final pseudo-cycle and 2. Damped cells are arrhythmic and have the largest amplitudes in their final pseudo-cycle.

For most simulations in the paper, we defined the smallest 30% (n = 228) of the oscillatory cells as weak and the remaining 70% (n = 595) as strong. For simulations that needed to distinguish between flat and damped, we chose the cut-off so there would be the same number of damped cells and weak cells.

### Model of SCN coupling and network topology

Model cells were coupled together by VIP signaling, simulated as a drive on the rate of *Per* transcription, as previously published [21]. In our model, 20 percent of the 400 neurons were capable of sending a VIP signal and all neurons could respond to VIP. Connections between cells were organized with a small world network topology as in [22] where each VIP cell was coupled to its four nearest neighbors and then had a probability of sending unidirectional long-range connections to other cells in the network. We set the connection probability to p = 0.05, resulting in a synchronized system with a range of 4 to ∼40 outgoing connections in most networks. To mimic the TTX experiments, we simulated 6 days with VIP-mediated coupling followed by 6 days with coupling eliminated and then reinstated for 6 days. We assessed the intrinsic circadian expression of each cell as well as the rate of resynchronization of each cell and the ensemble. The network connections and parameter values for each cell did not change throughout the simulation.

### Calculation of Sync Index

The synchronization index (SI) provides a real-time measure of the phase dispersion across a population of oscillators, which ranges from 1 (all cells peak in phase) to 0 (all cells peak at uniformly-distributed times of the day). We defined SI at each time *t* by the radius *r* of the complex order parameter [53] according to
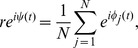
where *N* is the number of cells, *φ_j_(t)* is the phase of the *j^th^* cell at time *t*, and *ψ(t)* is the average phase of all cells. We compute the instantaneous phase of each cell (simulated or real) by applying the continuous wavelet transform using a Morlet wavelet [54] to its trace of *Period* mRNA. The phase of the cell over time may be recovered from the ridges of the transform, which are extracted using a straight-forward algorithm ([52], [54]; Wavos Package). Briefly, the continuous wavelet transform (CWT) produces a complex-valued field over scales (which may be mapped to instantaneous frequency) and translations (which may be mapped to time). The magnitude of the complex number at a given translation and scale may be interpreted as the strength of oscillation of the signal at the frequency given by the scale and the time given by the translation. The phase of the complex number at a given translation and scale gives the phase of that oscillatory component. By selecting points with contiguous scales across a range of translations that maximize the magnitude of the CWT (the “wavelet ridge”), we may extract the dominant frequency of the oscillator over time, and from those points extract the phase evolution of the oscillator from the angles of the CWT coefficients.

Because the wavelet analysis requires a window (in time) around the model state in question, it is unable to calculate the phase during the first and last 34 hours of each simulation. We treat each experimental condition separately, which mean there are gaps in the SI plotted in Fig. 3.

## Supporting Information

Figure S1 **Small changes in parameter values of a mechanistic model produce a range of oscillatory behaviors.** We sought to determine whether certain molecular events were more likely to contribute to the range of circadian phenotypes observed. Using a minimization algorithm we arrange each simulated cell (small box), representing one of 1296 potential combinations of parameters for transcription, translation, phosphorylation, and degradation of Period varied over 6 equivalent steps, to generate a two-dimensional visualization of multi-dimensional parameter space. This non-biased algorithm [36] groups cells into distinct areas of strong (blue), weak (cyan) and arrhythmic (black) outputs and ranking the contribution of each of these four parameters to rhythm generation. Axes for small boxes are determined by the lower order or less sensitive parameters, and these are then assembled to form larger, 6×6 grids, which vary with the higher order parameters. Rates for transcription and degradation appear on the larger axes, indicating that these are higher order parameters whose values produce a more dramatic shift in phenotype when changed. The red arrows emphasize how in three small steps within parameter space output transitions from arrhythmic to weak to strong, sustained circadian oscillations. While populations of strong and arrhythmic cells cover large ranges of parameters values, the number of weak cells at the transitions between these groups is much smaller.
(EPS)

Figure S2 **Parameter values in the mechanistic model straddle bifurcations, producing a spectrum of oscillator types.** (A) To produce a variety of circadian phenotypes in model cells, we probed parameters individually and selected ranges that produced a spectrum of behaviors when all other values were set to their published basal levels (with the exception of the maximal rate of per transcription, which is moved to 1.01). Here we show how the cumulative oscillator amplitude changes from strong (blue) to weak (cyan) to arrhythmic (gray) as a function of parameter value for the transcription, translation, phosphorylation and degradation of Per (left) and Bmal1 (right). (B) To determine whether another set of parameters produced similar phenotypes, we varied rates of transcription of *Bmal1*, or translation, phosphorylation and degradation of BMAL1 protein. Each plot visualizes areas of parameter space found by varying rates of transcription and translation (x-axis), phosphorylation, and degradation (y-axis) for both Period (left, replotted from Supp. Figure 1) and Bmal1 parameter sets (right), respectively.
(EPS)

Figure S3 **The mechanistic model produces cells with a spectrum of period and amplitude values consistent with SCN data.** When we examined the period (left) and cumulative oscillator amplitude (right) values of simulated cells, here plotted using the same axes as Supp. Figs. 1 and 2, we found that cells falling in the region of parameter space corresponding to weak oscillators show a range of period values (18 to 30 h) similar to what was observed in functionally isolated SCN neurons [10]. The range of final amplitude values of weak cells was lower than that of strong cells (range 2–10 arbitrary units vs. range 12–28 a.u.). We hypothesized that a heterogeneous mix of oscillators with periods and amplitudes that are easily tuned are beneficial to resynchrony.
(EPS)

Figure S4 **Intrinsic amplitude and period do not correlate.** We plot the intrinsic period against the intrinsic amplitude (normalized to the largest value) for slice 1 (A), slice 2 (B), and the model (C). The model tissue is composed of 80% weak and 20% strong cells. The slice amplitude is measured as the amplitude during the final day of TTX treatment and its period is the mean value of the Wavos-computed period throughout TTX treatment. In all cases, there is similar period variability and the period and amplitude are un-correlated.
(EPS)

Figure S5 **Three 100-cell networks comprised of 50% sustained cells (blue) and 50% weak cells (cyan).** All are connected using a small world network, but with different classes of cells at the “hubs” - (**A**) sustained cells are placed at hubs, (**B**) no cell class is given preference, and (**C**) weak cells are placed at hubs. The size of the cell is proportional to the number of cells it connects to. Most network connections are shown in light gray. One “hub” cell is highlighted in each network, and its connections are dark gray.
(EPS)

Figure S6 **Damped and weak cells have similar effects on the network.** We show heat plots of the quality of synchrony after the wash for varying demographics (**A**) and varying types of cells at network hubs when 50% of the cells are strong (**B**), with red indicating perfect synchrony and blue indicating no synchrony. The y-axis of both plots indicates the fraction of non-strong (i.e. weak and damped) cells that is weak. The top row in both plots corresponds to the data in Figure 3. In (**A**), we show that when there are no strong cells (leftmost column), synchrony is high, regardless of whether the population is composed of all damped cells (bottom), a mix of damped and weak cells (middle), or all weak cells (top). For each column, the color remains mostly constant, indicating that the quality of sync is a function of the fraction of oscillators that are strong, and that weak and damped oscillators are interchangeable. In (**B**), we show that placing weak or damped cells at the hubs allows for better synchrony than placing mixed or strong cells at the hubs. Note that placing weak cells at hubs in populations with no weak cells is equivalent to placing mixed cells at hubs. Likewise, placing damped cells at hubs when there are no damped cells is equivalent to placing mixed cells at hubs.
(EPS)

Text S1 **Statistical analysis.** We examine two SCN explants and corresponding simulation data for evidence that intrinsic properties of cells predict emergent behaviors in coupled conditions. We find no correlation between any intrinsic property and a cell's behavior after coupling has been established, for either the model or the explants. For the explants, however, we find evidence of a spatial pattern, in which weak, lateral cells phase lead strong, medial cells.
(PDF)

Text S2 **Reduced model details.** We define the reduced model and demonstrate that it accurately captures the behavior of the mechanistic model used in the main text.
(PDF)
