# Supplementary material for: Weakly Circadian Cells Improve Resynchrony
Source: PLoS Comput Biol. 2012 Nov 29;8(11):e1002787. doi: 10.1371/journal.pcbi.1002787 (PMC3510091; doi:10.1371/journal.pcbi.1002787)
Supplement: Text S1 — Statistical analysis. We examine two SCN explants and corresponding simulation data for evidence that intrinsic properties of cells predict emergent behaviors in coupled conditions. We find no correlation between any intrinsic property and a cell's behavior after coupling has been established, for either the model or the explants. For the explants, however, we find evidence of a spatial pattern, in which weak, lateral cells phase lead strong, medial cells. (PDF) [file pcbi.1002787.s007.pdf]

Text S1: Statistical Analysis (Webb et al.)

Here we examine two SCN explants and corresponding simulation data for evidence that intrinsic properties of cells predict emergent behaviors in coupled conditions. We find no correlation between any intrinsic property and a cell's behavior after coupling has been established, for either the model or the explants. For the explants, however, we find evidence of a spatial pattern, in which weak, lateral cells phase lead strong, medial cells.

The document is organized as follows:

1. We identify 9 intrinsic properties of the cells, calculated from data collected in uncoupled (TTX) condition. We compare the distributions of the model and biological data properties and find they are qualitatively and quantitatively similar.
2. We identify 2 emergent, tissue behaviors of the cells in the network, calculated from data collected in the re-coupled (wash) condition. We compare the distributions of the model and biological data properties and find they are qualitatively and quantitatively similar. We also include an analysis of cells that are phase-leading or phase-lagging in the coupled tissue.
3. Treating the properties and behaviors as continuous variables, we pair each property with each behavior and find no (univariate) correlation for either the explants or the model.
4. Treating the properties and behaviors as continuous variables, we examined the ability of a linear combination of the features to predict either behavior (multiple linear regression). The analysis revealed no suitable combination of features for either the explants or the model.
5. Treating the behaviors and positions as discrete (category) variables, we perform an ANOVA and learn that, for the explants there are significant differences in amplitude and phase between laterally located and medially located cells. We find no statistically significant patterns in the model data.

1. Computing intrinsic properties (in isolation)

The intrinsic properties are computed from cell traces during the 6-day TTX treatment. For the explant data, we used the bioluminescence trace over time. For the simulation, we used the levels of Per mRNA over time. The position of each cell is simply its location during the recording. The first 4 properties provide measures of abundance, the next two of (peak-to-trough) amplitude, the next two of period, and the final property is a measure of position. The properties are:

1. Mean Per abundance (as measured by bioluminescence levels) throughout TTX
2. Mean Per abundance on day 6 of TTX
3. Per abundance in the final hour of TTX
4. Mean Wavos-computed power throughout TTX
5. Intrinsic amplitude computed as the peak-to-trough amplitude of per on day 6 of TTX
6. Peak-to-trough amplitude of per on day 6 of TTX, relative to the peak-to-trough amplitude of per on day 1 of TTX
7. Mean (Wavos-computed) period throughout TTX
8. Intrinsic period. For the explant data, this is the mean (Wavos-computed) period of the last 24 Wavos-detectable hours of TTX treatment. For the simulation, it is the period computed once the cell has reached its limit cycle.
9. Cell's (Euclidean) distance from the center of the nuclei. For the explants, we use different centers for each nucleus, so that the cells along the outer edge of each nucleus have the same distance. For the simulation, we assume the cells are laid out in a regular 2-dimensional grid, and compute the distance from the center of the grid.

1.a. Spatial information

In Figure S7, we demonstrate the distance-from-center measure. Earlier analyses led us to believe that, after the washout of TTX, there was a spatial pattern in the phase of cells across an explant. This wave appears to move from the lateral to medial positions. To confirm or reject the presence of the wave, we need a measure that differentiates lateral cells from medial cells. Thus, we

compute the distance from center of the two nuclei.[1] We analyzed the pair of bilateral nuclei in slice 1, only the right nucleus in slice 2, and we treat the model as one nucleus.

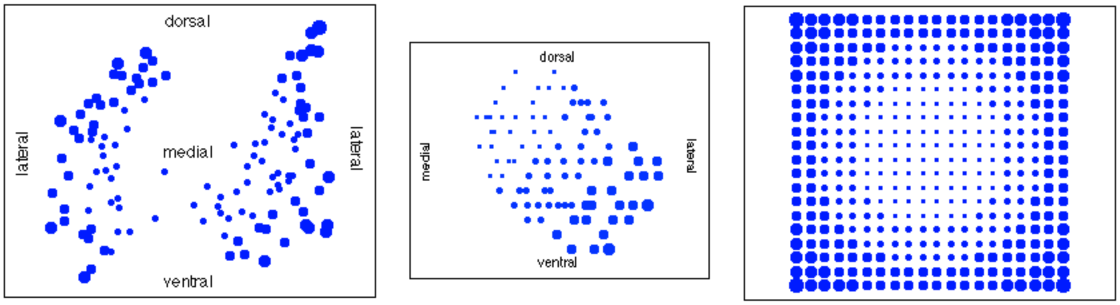

Figure S7. Demonstration of the distance-from-center measure. We demonstrate the distance-from-center measure for slice 1(left), slice 2 (middle), and a simulation (right). Each circle is a cell, and the size of the circle indicates the distance of the cell from the center of the SCN.

1.b. Distributions of explant cell properties

In Figure S8 and Figure S9, we show the distributions of all slice 1 and slice 2 cell properties, respectively. The properties of cells in the slices are similar. We find all measures of amplitude indicate that smaller oscillations dominate and the period measures indicate qualitatively normal distributions with means of approximately 24 h (slice 1) or 26 h (slice 2) and standard deviations of approximately 2 h (both slices).

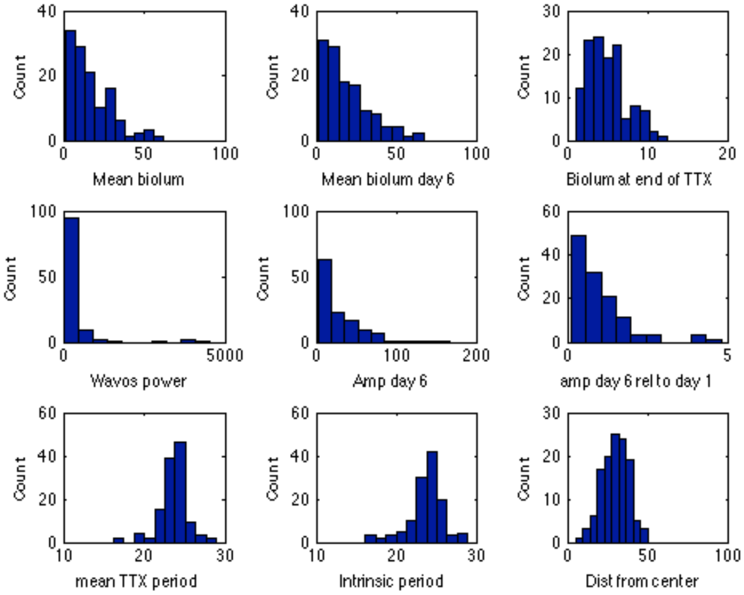

Figure S8. Histograms of slice 1 cell properties show that amplitude properties follow exponential distributions while period and distance-from-center properties follow normal distributions. The top two rows have measures related to amplitude. The bottom row has two period measures and a position measure.

---

[1] Because the two nuclei are tilted at slightly different angles, we actually use a different estimate of "center" for each nucleus.

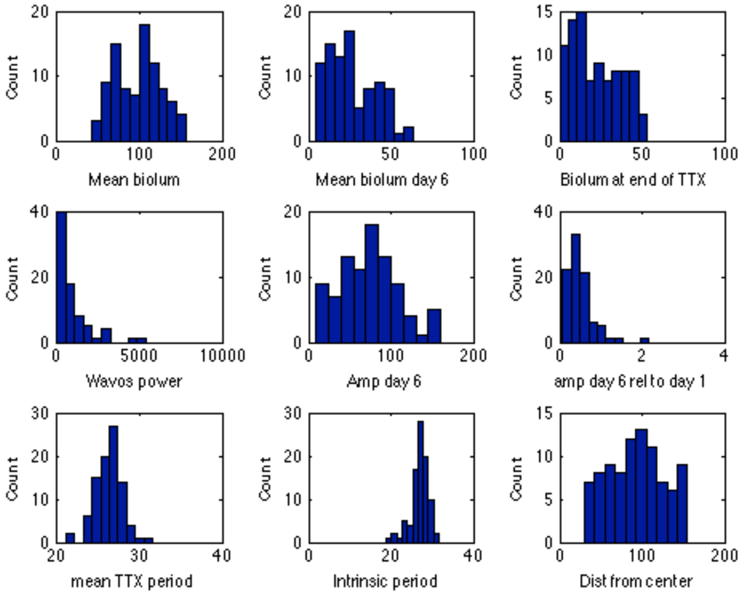

Figure S9. Histograms of slice 2 cell properties show that amplitude properties favor small values while period and distance-from-center measures are approximately normal. The top two rows have measures related to amplitude. Two amplitude measures follow exponential distributions (power and relative amplitude). The remaining amplitude measures follow qualitatively normal distributions with positive skew. The bottom row has two period and a position measure.

1.d. Distributions of simulated cell properties

In Figure S10, we show the distributions of all simulated cell properties. We use a simulation with 80% weak cells and 20% strong cells.

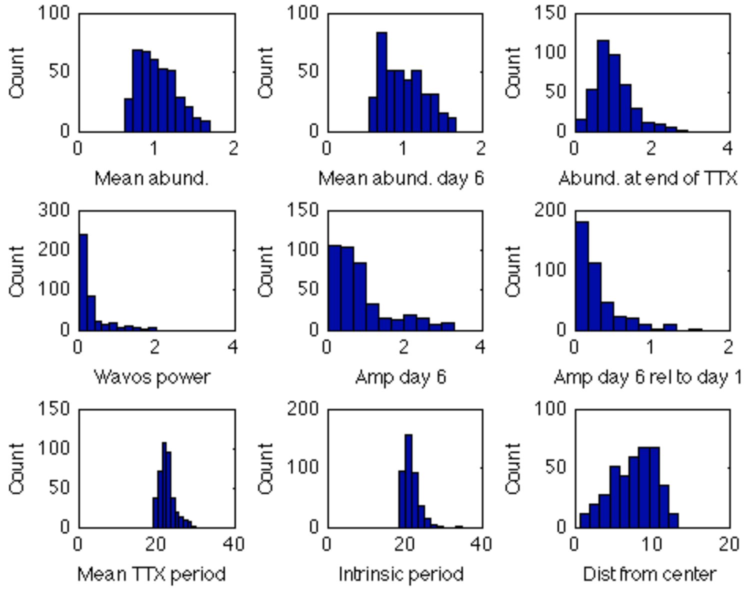

Figure S10. Histograms of model cell properties show that most amplitude properties follow exponential distributions while period and distance-from-center properties follow normal distributions. The top two rows have measures related to amplitude. The bottom row has two period measures and a position measure.

For both of the explants and the simulations, the amplitude measures follow an exponential distribution or are skewed toward small amplitudes and the period measures follow a normal distribution. The only difference is that the mean abundance values for the explant data follow an exponential distribution whereas the simulated data do not.

2. Computing cell behaviors (in tissue)

The emergent behaviors are computed from 6-day long cell traces after TTX has been washed out. There are two behaviors:

1. Entrance time after the wash (this is the hour at which Wavos first detects a phase for the cell).
2. Strength of phase-leadership. This is computed as the fraction of time a cell spends as a phase-leader (after the wash). At each time step for which at least 80% of the cells are rhythmic, we determine whether a cell is ahead of or behind the mean phase. Over time, a cell may transition from being a momentary leader to a momentary lagger. Thus a measure of phase-leadership is the fraction of time a cell spends as a momentary phase leader. Maximal strength indicates the cell is always a phase leader. Minimal strength indicates the cell is always a phase lagger.

For both of these behaviors, we treat them as continuous variables (shown above) and as discrete (category) variables:

1. Entrance time cohorts.
   a. For slice 1, cells in cohort 1 become rhythmic first (hours 31-35, n=39), cells in cohort 2 become rhythmic second (hours 36-45, n=43), and cells in cohort 3 become rhythmic after hour 46 of the wash (n=32). (Note: these are the same cohorts shown in Figure 1 in the main text).

b. For slice 2, we are missing the first 48 hours of the wash, so most cells have already become rhythmic by the time Wavos can detect them. Cells in cohort 1 become rhythmic first (28-35 hours after the recording begins, n=51), cohort 2 becomes rhythmic second (hours 36-40, n=23), and cells on cohort 2 become rhythmic after hours 46 of the wash (n=15).
c. For the simulated data, there is only one cohort, because most cells become rhythmic at the same time.

2. Phase leader groups. Cells in the phase-leader group spend most of their time with phases close to and ahead of the mean phase, cells in the phase-lagger group spend most of their time close to and behind the mean phase, cells in the "drifting" group spend most of their time close to the mean phase and transition between leading and lagging, and cells in the "non-participant" group are not close to the mean phase.

Below, we describe the properties of the explant and simulations. We follow it with an analysis of the difference between the explant and simulations.

2.a. Entrance times in the explants

We show the bioluminescence traces of slice 1, according to the entrance time cohort in Figure S11 These are the same cohorts as shown in Figure 1 of the main text. We show both the mean traces (top subfigure), and then all cells within each cohort. The first cohort clearly shows rhythms earlier than cohorts 2 and 3. The peak of the mean cohort 1 trace is earlier than the peak of the mean cohort 2 trace, and it appears that the mean cohort 1 trace is, in general, phase-leading the mean trace of cohort 2. Cohort 3 shows less organization.

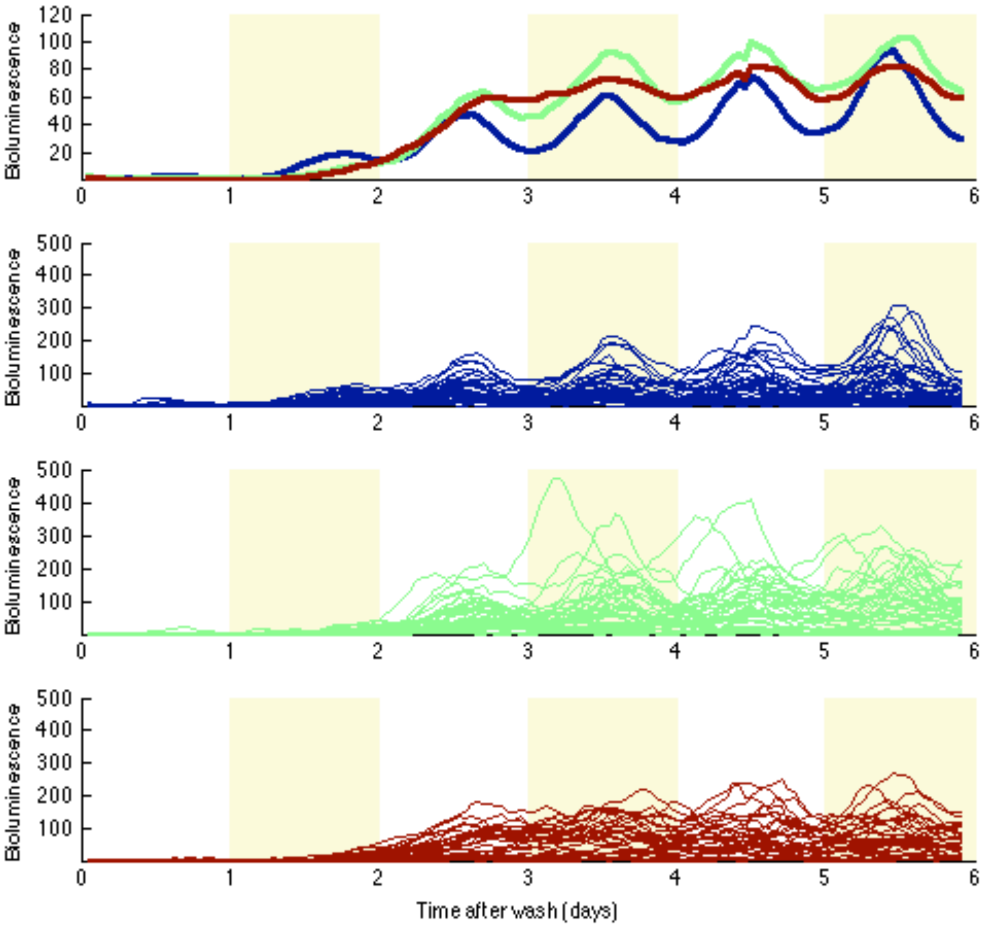

Figure S11. The mean bioluminescence of each entrance cohort in slice 1 represents its overall collective behavior. We show bioluminescence traces after TTX is washed out of the explant. On the top, we show the mean traces (determined by entrance time, blue enters first, green second, red third). The remaining sub-figures show all cells, color-coded by cohort. For ease of comparison, we shade days 2, 4, and 6.

A similar analysis of slice 2 does not produce as clear of a result. Because the first two days of wash data are missing, we begin our analysis on day 3, and all cells have regained rhythmicity by then (data not shown). Instead, we group the cells in slice 2 according to region, and see that lateral cells are lower in amplitude and phase-lead medial cells (Figure S12).

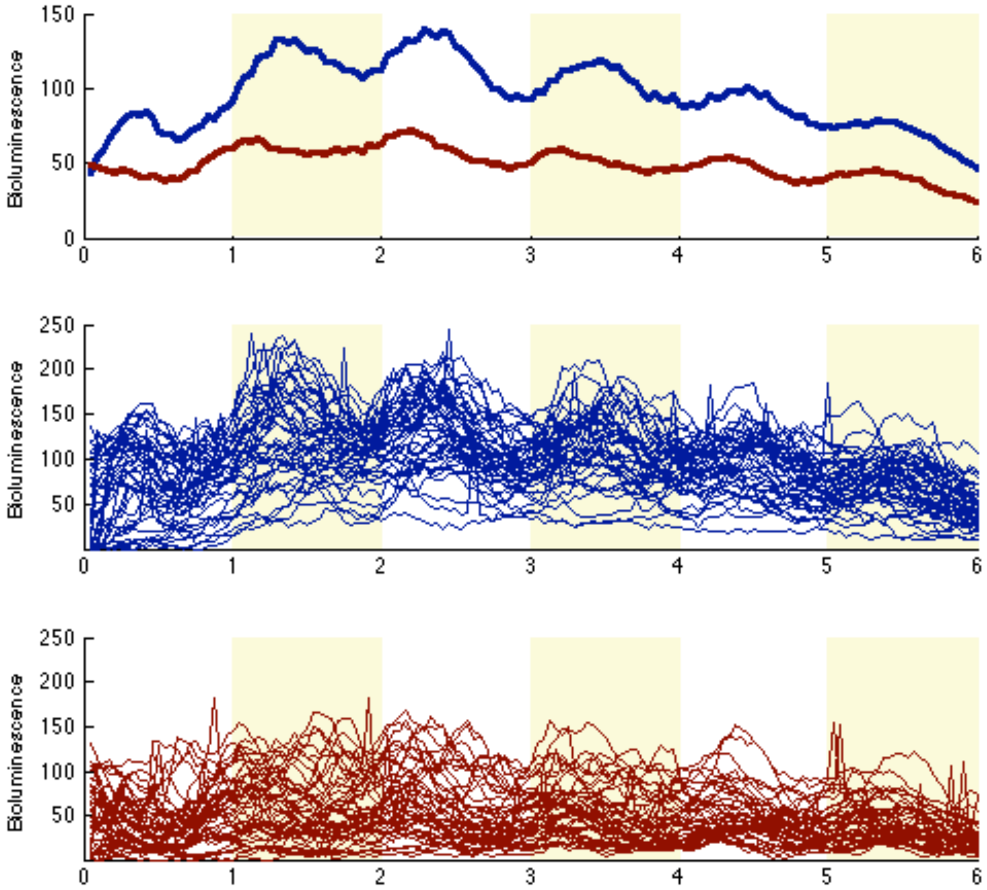

Figure S12. The mean bioluminescence of each region in slice 2 represents its overall collective behavior. We show bioluminescence traces after TTX is washed out of the explant. On the top, we show the mean traces (determined by region, blue is medial, red is lateral). The remaining sub-figures show all cells, color-coded by region. For ease of comparison, we shade days 2, 4, and 6.

2.b. Details about phase-leadership computation

We are interested in learning about which cells lead and lag when there is at least a moderate level of synchrony so we consider points in time when the synchronization index is greater than or equal to 0.5. We compute the mean phase at each qualifying time point in the wash and compare the phase of each cell to it. If a cell is within 1/6 of a cycle of the mean phase and is ahead, we consider it a momentary leader. If it is within 1/6 of a cycle of the mean and is behind, we consider it a momentary lagger. We then compute the fraction of time a cell spends as a momentary leader.[2] A cell is classified as a leader if it spends more than 80% of its time as a momentary leader and as a lagger if it spends more than 80% of its time as a momentary lagger. A cell is classified as drifting if it transitions between being a phase-leader and a phase-lagger and as a non-participant if its phase is not computable or is consistently more than 1/6 of a cycle away from the mean phase.

[2] The denominator is the total amount of time spent as either a momentary leader or as a momentary lagger. Time spent more than 1/6 of a cycle away from the mean phase doesn't count. We chose to include only those cells close to the mean under the assumption that cells too far away aren't part of the pattern (i.e. we am looking at a phase wave, and the cells participating in the phase wave have been properly reined in).

2.c. Phase-leadership: Analyzing the explants

In Figure S13, we plot the result applied to the slice 1 as histogram. The data for slice 2 are similar (data not shown).

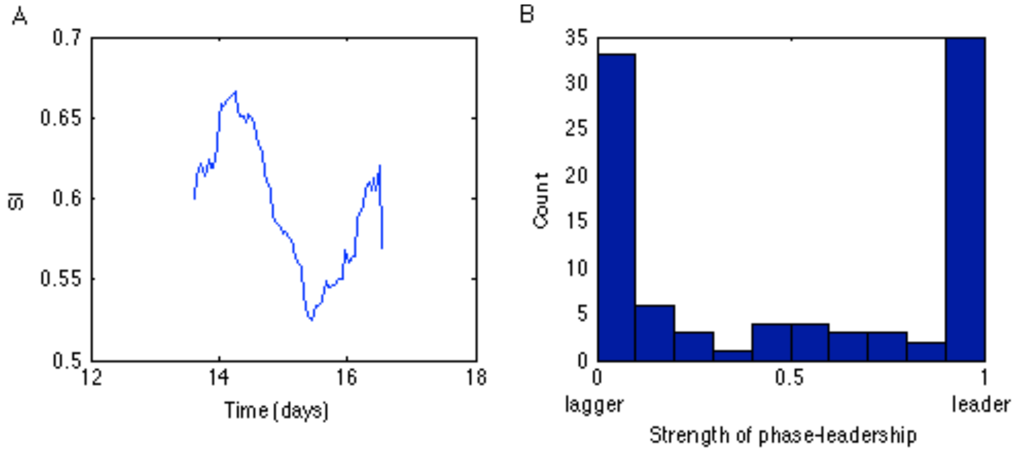

Figure S13. Most cells in slice 1 are either phase-leaders or phase-laggers. For reference, we show the synchronization index (SI) for the cells after the wash (A). In (B), we show the strength of phase-leadership, measured by the fraction of time a cell spends as a momentary phase leader over the time spent near the mean phase. Most cells have values close to 0 or 1, indicating they can be classified as either phase-leaders or phase-laggers.

2.d. Phase-leadership: Analyzing simulations

We performed a similar analysis of the simulated populations and found similar results (data not shown). We observed that for populations with higher synchrony, there are more cells that are always leading or always lagging. When populations fail to synchronize, some cells spend some time as leaders and some time as laggers.

3. There is no univariate correlation

The goal of this study is to determine if any intrinsic properties explain network behavior in the wash. A simple test for this relationship is to correlate each of the 9 cellular properties with each of the 2 emergent tissue-level behaviors. We add the distance-from-center to the list of behaviors (so it serves as both an independent and a response variable). It also allows us to search for a spatial pattern in the other properties. Thus, we pair each of 9 independent variables with each of 3 dependent variables (excluding the pair that would have distance as both the independent and dependent variables). For each pair, we computed the correlation coefficient between the property and the behavior, between the natural log of the property and the behavior, and between the property and the natural log of the behavior. There was no correlation ($R^2 < 0.2$) for any of the pairs, with the exception of weak correlation ($0.2 < R^2 < 0.25$) for amplitude and distance-from-center in slice 2 (indicating that lateral cells had smaller oscillations). We verified that there were no other clear patterns by visually inspecting the scatter plots. This was true for the explants and for simulations. In Table S1, we show the largest R^2 value for each explant and simulation examined.

Data Set	Maximum R^2
Slice 1	0.07
Slice 2	0.25
simulation (80% weak, 20% strong, mixed hubs)	0.08
simulation (90% weak, 10% strong, mixed hubs)	0.15
simulation (100% weak)	0.05

Table S1. Maximum correlation coefficients for univariate analysis of explant and simulated data.

4. A linear combination of the properties fails to predict the behaviors

Individually, intrinsic properties fail to correlate with the behaviors. But that does not exclude the possibility that a combination of the properties can predict the behaviors. We test the hypothesis that a linear combination of the properties correlates with the behaviors. For each of the two behaviors, we perform a multiple linear regression with the set of 9 features. For both the data and the simulation, we find no correlation.

For the explants, the results are immediately clear. We perform the linear regression, find a least-squares solution, and use it to predict the behavior under consideration. There is no correlation between the predicted behavior and the observed behavior ($R^2 = 0.11$ for entrance time and $R^2 = 0.1$ for strength of phase-leadership). Further, we fail to reject the null hypothesis that a constant value is a better predictor than the set of features ($p > 0.05$).

For the simulation (80% weak, 20% strong, mixed cells at hubs), the results are similar, but a little less straightforward. We can reject the null hypothesis ($p < 0.05$). Statistically, the linear combination is significant. However, the linear predictions are not informative ($R^2 = 0.23$ for entrance time and $R^2 = 0.2$ for strength of phase-leadership). Thus, we conclude that a linear combination of the features fails to predict emergent behaviors in both the model and the explant.

5. Treating the behaviors as category variables reveals subtle patterns in the explant

There is no continuous dependence of the behaviors on the properties (in that there is no correlation), but that does not rule out the possibility that there are differences in properties if we group the cells according to behavior or position. Here, we describe our 1-way ANOVA analysis of cell properties and behaviors. We find subtle spatial relationships.

5.a. ANOVA analysis of the explant

We define 3 category variables:

1. Position: The 50% of the cells with the smallest distance from the SCN centers are categorized as medial, and the remaining 50% as lateral
2. Entrance Time Cohort: We use the 3 cohorts, based on entrance time as described in Section 2
3. Phase leader/lagger: Cells that are momentary phase leaders 80% of the time or more are categorized as leaders. Cells that are momentary phase laggers 80% of the time or more are categorized as laggers. (Again, this is as described in Section 2.)

We use the 9 properties described in Section 1. To ensure the amplitude-related properties follow a normal distribution, we perform our analysis with the natural log of the amplitudes. By visual inspection, we verified that the 9 properties (with the log transformation, where applicable) followed normal distributions.

For each slice, for each pair of continuous and category variables, we perform a 1-way ANOVA. For the first 9 rows, we use Matlab's ANOVA1 function. The ANOVA results indicate that the null hypothesis (of all groups having the same mean) can be rejected ($p<0.05$) for a small number of property/behavior pairs. In Table S2, there is an entry for each pair for which we rejected the null hypothesis. The contents of the entry provide information about that difference. For two of the columns there were only two groups. For the entrance cohort variable, there are 3 groups. To determine which groups have significantly different means (with a 95% confidence interval), we perform a multiple comparison procedure (Tukey-Kramer HSV) using the Matlab function multcompare.

	Medial vs. Lateral	Entrance cohort	Phase leader vs. lagger
ln(mean biolum)	Slice 2: Medial cells are higher amplitude than lateral cells.		
ln(mean biolum day 6)	Slices 1 & 2: Medial cells are higher amplitude than lateral cells.		
ln(biolum at end of TTX)	Slice 2: Medial cells are higher amplitude than lateral cells.		
ln(amp day 6)	Slices 1 & 2: Medial cells are higher amplitude than lateral cells.	Slice 2: Cohort 2 cells are larger than cohort 3 cells.	
ln(amp day 6 rel to day 1)	Slices 1 & 2: Medial cells are higher amplitude than lateral cells.	Slice 1: Cohort 1 cells are smaller than cohort 2 cells.	
ln(Wavos power)	Slice 2: Medial cells are higher amplitude than lateral cells.		
Mean TTX period	Slice 2: Medial cells are faster than lateral cells.		
Intrinsic period	Slice 2: Medial cells are faster than lateral cells.		
Distance from center	Medial cells are closer to the center than lateral, by definition.	Slice 1: Cohort 1 cells are further from the center than either cohort 2 or cohort 3 cells.	Slice 2: Phase leaders are further from the center (more lateral) than phase laggers.

Table S2. Results of 1-way ANOVA analysis of both explants reveal relationships between distance-from-center, phase-leadership, and amplitude. Each row represents a continuous variable (or set of them). Each column represents a category variable. The continuous variables include all 9 properties and the momentary phase-lead or lag of each cell. Entries are non-empty when the difference in means is significant ($p < 0.05$). The entry begins by identifying the slice(s) for which the difference is significant and then provide a qualitative description of the difference.

The results are consistent with the presence of a spatial pattern in the phase behaviors. They are not identical for the two slices, but in all cases for which both slices had significant differences, those differences were the same. For both slices, there is a spatial pattern suggested in which lateral cells are lower in amplitude and act as phase-leaders.

5.b. ANOVA analysis of the simulations

We analyze simulation data for three different network configurations with mixed cell types at hubs: (1) 80% weak, 20% strong (2) 90% weak, 10% strong, and (3) 100% weak cells. We find no significant evidence of spatial effects.[3] There is, however, weak evidence for period effects, in that

[3] The only statistically significant difference between outer (corresponding to lateral cells in the slice) and inner (corresponding to medial cells in the slice) cells was for the simulation with 100% weak oscillators. The mean log of the abundance in the final hour of TTX was -0.023 ln(a.u.) for the inner cells and -0.106 ln(a.u.) for the outer cells.

all three configurations have phase leaders with faster periods (using the mean period during TTX) and that one configuration (100% weak cells) has phase leaders with faster periods (using the intrinsic period measure).

5.c. Conclusions

The model and explants have clearly different behaviors. Most of this difference can be attributed to spatial organization in the explants. It is interesting that the model suggests that faster cells are phase leaders. This is consistent with our intuition and with results from other modeling studies (e.g. Hafner et al., 2012). We found no such relationship in the explant data (and in fact, we found the opposite for slice 2). One possibility is that the data were not collected with high enough temporal precision to extract a sufficiently accurate measure of period. Another possibility is that spatial differences in the network caused large enough phase differences that any within group differences caused by period differences were masked. This is a subject of future research.
