# Supplementary material for: Weakly Circadian Cells Improve Resynchrony
Source: PLoS Comput Biol. 2012 Nov 29;8(11):e1002787. doi: 10.1371/journal.pcbi.1002787 (PMC3510091; doi:10.1371/journal.pcbi.1002787)
Supplement: Text S2 — Reduced model details. We define the reduced model and demonstrate that it accurately captures the behavior of the mechanistic model used in the main text. (PDF) [file pcbi.1002787.s008.pdf]

Text S2: Reduced Model Details (Webb et al.)

Phase-based Reduced Model

We designed a reduced model to mimic the mechanistic model used in the main text. The model for each cell was developed by Leloup &Goldbeter (2003) and augmented to simulate a coupled population of cells by To et al. (2007) and later by Vasalou et al. (2009). We call this model the "full model" or the "mechanistic model". There are three properties of the mechanistic model in which we are most interested: the trace of per mRNA, the VRC associated with the maximal rate of per mRNA transcription (vsP), and the trace of VIP release. We sought to create a simplified model that would mimic the amplitude changes, period changes, phase response, and signaling behaviors of the mechanistic model. If our simplified model cells could be placed in a network and demonstrate the same emergent behaviors as the full model, then we could gain confidence in our analysis of the full model, namely that we had focused on the appropriate set of characteristics.

We designed the reduced model to recapitulate these features of the full population model:

1) Lower amplitude oscillators have higher magnitude vsP VRCs.
2) Oscillators have higher amplitudes when coupled than when uncoupled. Thus, signaling increases the amplitude.
3) Different oscillators relax to different steady-state amplitudes.
4) The signal an oscillator sends is proportional to its per mRNA levels.
5) VIP signals affect oscillators ultimately by increasing the value of vsP. In other words, the speed adjustments are controlled by the vsP VRC.

To construct a reduced model capturing the above aspects, we require

1) A means of tracking and adjusting the phase
2) A means of tracking and adjusting the amplitude
3) A means of sending and receiving signals

We use two ordinary differential equations to capture the phase and amplitude values for each oscillator. The model is a generic 2D limit cycle oscillator in polar coordinates with two key additions: 1) the amplitude affects the phase response and 2) the amplitude responds directly to the signal.

$$\frac{d\phi_i(t)}{dt} = 1 + \mathrm{VRC}(\phi_i(t), A_i(t), ss_i, \tau_i)\frac{\gamma_i(t)}{K + \gamma_i(t)}$$

$$\frac{dA_i(t)}{dt} = -\lambda(A_i(t) - ss_i) + \kappa\frac{\gamma_i(t)}{K + \gamma_i(t)}$$

where ϕ_i is the phase at time t, $A_i(t)$ is the amplitude (in a.u.) at time t. Their dynamics depend on a VRC, the concentration γ_i of VIP at cell i's receptors (i.e, the signal), the cell amplitude's decay rate λ, and the cell amplitude's growth rate κ. The concentration of VIP saturates, with an activation threshold K. Note that the VRC is a function of the phase, the current amplitude, the steady-state amplitude (ss_i), and τ_i, which is the intrinsic period of the cell.

Here are more details:

1) The phase ODE is the first part of a 2D oscillator in polar coordinates. It is also a standard "phase reduction" or "phase-only" model. Phase progresses with time, unless there is a signal. When there is a signal (i.e. γ_i /= 0), the phase velocity speeds up or slows down according to the response curve (here, it is the VRC).
2) The VRC function mimics a vsP VRC computed from the full model. It is naturally a function of phase. It is also a function of steady-state amplitude (ss_i) – smaller ss_i amplifies the change in phase velocity while larger ss_i reduces the change. We have measured this relationship for the mechanistic model (see Figure S15). We assume that the current amplitude has a similar effect. Although this is not easily measured in a limit cycle model, it is intuitive. Consider a radial isochron clock like that in the Poincare oscillator (Glass & Mackey, 1988). Near the center of the oscillation, isochrons are "close" together and a small perturbation will easily move the trajectory from one isochron to another. But far from the center, these same isochrons are further apart and the same perturbation (in terms of x-y distance) will move the trajectory across fewer isochrons. Small amplitude oscillators have trajectories closer to the center and large amplitude oscillators have trajectories further from the center. Thus small amplitude oscillators are more shiftable than large amplitude oscillators.
3) The amplitude ODE is also built on a standard 2D oscillator model. When no signal is present (and therefore γ_i is 0), the amplitude approaches its steady-state (ss_i). It does so with a decay rate lambda. When there is signal, the amplitude undergoes a transient increase. This mimics the rapid response of per mRNA levels to VIP application.
4) The local VIP concentration seen by each cell i is the average concentration of VIP sent by each cell that is connected to cell i. The network topology is a small world network, as modeled by Vasalou et al. Suppose there are NS_i cells sending VIP to cell i. We average the signal sent by each of those cells (using j as the index):
$$\gamma_i(t) = \frac{1}{NS_i}\sum_j VIP_{\text{sent by } j}(t)$$
5) The VIP concentration sent by each cell has a profile similar to that of a saturated per mRNA. This is similar to that computed by To et al and Vasalou et al. (where VIP release is a function of per mRNA). Our version allows for VIP release to be out of phase with per mRNA up to four hours:
$$VIP_{\text{sent by } i}(t) = 12\frac{perLike_i(t)}{4 + perLike_i(t)}$$
Since no per-like species is explicitly modeled, we compute it using the current phase and amplitude of the oscillator. The trace of per mRNA in the LG-based model is nearly sinusoidal with a peak approximately one quarter of the way through the cycle. The per-like species peaks within 4 hours of the per mRNA peak:
$$perLike_i(t) = \frac{A_i(t)}{2}\sin\left((\phi_i(t) - \delta)\frac{2\pi}{\tau_i}\right) + \frac{A_i(t)}{2}$$
where δ is the difference in phase between the per mRNA peak and the peak of the per-like species. δ is in the range -4 to 4.
6) We estimate per mRNA according to
$$perMRNA_i(t) = \frac{A_i(t)}{2}\sin\left(\phi_i(t)\frac{2\pi}{\tau_i}\right) + \frac{A_i(t)}{2}$$
where τ_i is the intrinsic period of cell i.

A VRC that takes into account the amplitude

Each reduced model cell will mimic a specific full model cell. Each full model cell has a steady-state amplitude and an intrinsic period. Some have VRCs, but those VRCs are defined only at the steady-state amplitude. For cells without VRCs, we need to construct one. For all cells, we need to

adapt the VRC so that it attenuates when the cell's amplitude is higher than its steady-state amplitude. In this section, we describe how we construct VRCs for the reduced model using VRCs from the full model.

Define the VRC at steady-state amplitude

For all sustained and some damped full model cells, it is possible to compute a VRC to vsP. For corresponding cells in the reduced model, we simply use this VRC. It is a function of phase and the intrinsic period. This intrinsic VRC is computed at time points 6 minutes apart. When we need to know its value between time points, we use linear interpolation.

$$\mathrm{VRC}_{intrinsic}(\phi_i(t), ss_i, \tau_i)$$

For those cells without VRCs, we must construct one. The VRC function is constructed from a representative LG-based model vsP VRC, which is then scaled according to the steady-state amplitude. From the LG-base model, we chose a vsP VRC from a cell that has a per mRNA oscillation of amplitude 1 a.u. (see Figure S14). This cell is classified as sustained, but it is of relatively small amplitude. We scale the VRC magnitude according to the steady-state amplitude of the reduced model oscillator and scaling coefficients determined by an analysis of the LG-base model oscillators (see Figure S15), which shows the area under the VRC can be approximated by an exponential function of the steady-state amplitude.

We scale the reference VRC by computing the ratio between the areas of the VRC for cell i and the reference cell (which has a steady-state of 1):

$$\mathrm{VRC}_{intrinsic}(\phi_i(t), ss_i, \tau_i) = \frac{e^{-0.46269 ss_i}}{e^{-0.46269}} \mathrm{VRC}_{ref}(\phi_i(t), ss_i, \tau_i) = e^{0.46269(1-ss_i)} \mathrm{VRC}_{ref}(\phi_i(t), ss_i, \tau_i)$$

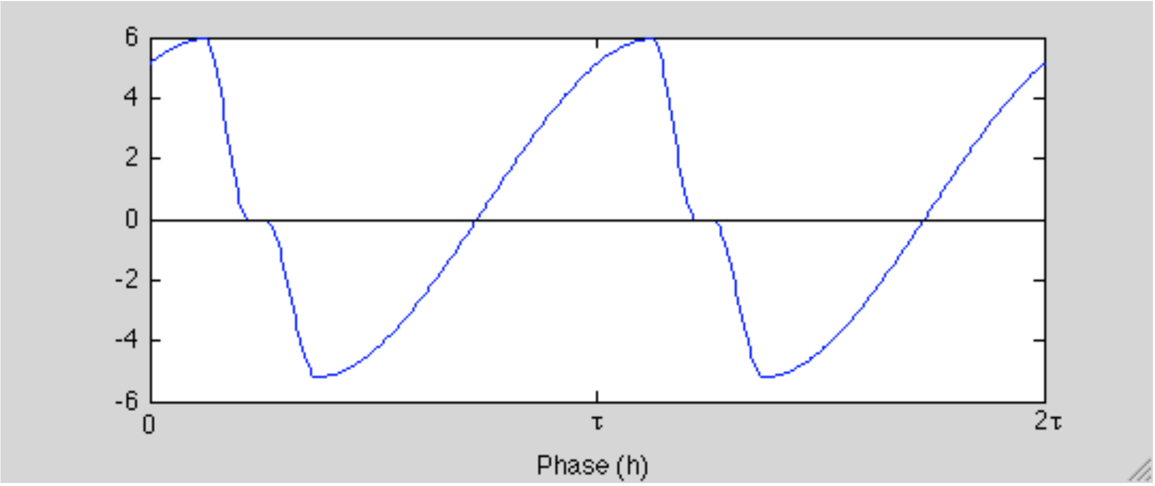

Figure S14. The VRC for a cell with amplitude 1a.u. is shown over two periods.

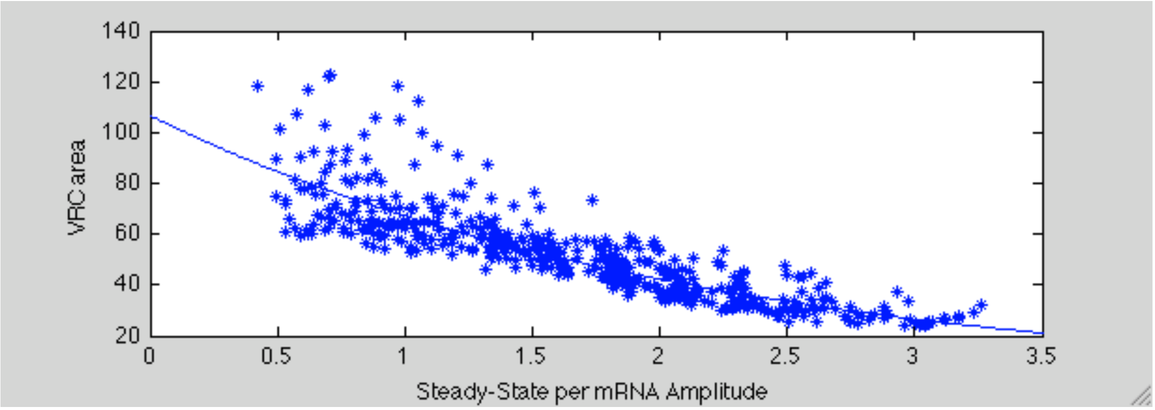

Figure S15. For each rhythmic cell in the LG-based model population, we compute a vsP VRC. We compute the area under the absolute value of the VRC and plot it against the steady-state amplitude of the per mRNA oscillations. Four outliers (with VRC areas less than 5 or greater than 180) are removed. The natural logarithm of the VRC area anti-correlates (R=-0.9) with the steady-state amplitude of per mRNA. The line of regression has slope -0.46269 and y-intercept 4.6691. The dependence of the VRC area on the steady-state is exponential.

Make the VRC dependent on the current cell amplitude

For both types of cells (those for which we have VRCs and those for which we needed to construct one), we need to explicitly model its dependence on the current amplitude. When a cell's amplitude is larger than its steady-state amplitude, its VRC is attenuated.

$$\mathrm{VRC}(\phi_i(t), A_i(t), ss_i, \tau_i) = e^{\beta(ss_i(t) - A_i(t))}\,\mathrm{VRC}_{intrinsic}(\phi_i(t), ss_i, \tau_i)$$

where β is a parameter we must identify. We expect it to be close to 0.463, which is the exponent for the dependence of the VRC on the steady-state amplitude.

Model Parameters

There are two parameters unique to each cell:

Parameter	Meaning
ss_i	steady-state peak-to-trough amplitude of cell i
τ_i	intrinsic period of cell i

There are four parameters whose value is identical for all cells:

Parameter	Meaning
λ	decay rate of peak-to-trough amplitude of a cell
κ	growth rate of peak-to-trough amplitude of a cell, in response to signal
K	activation threshold for VIP signal
δ	phase difference between peak of VIP and peak of per mRNA (a positive value for δ means VIP peaks later than mRNA).
β	determines the dependence of the VRC magnitude on the current amplitude

To simulate tissue with the phase model, we use ss_i, τ_i, and VRCs from taken from the full model. We optimize for the remaining parameters (λ, κ, K, δ, and β).

Parameter-Fitting Procedure

We use an evolutionary strategy to find appropriate parameter values for λ, κ, Κ, δ, and β. The cost function simulates a coupled model and assesses its ability to synchronize with a period and amplitude matching that of the full model. The simulation begins with the cells out of phase and with amplitudes equal to their steady-state amplitude. We use a small-world network of 400 cells, 80% of which are weak oscillators, 20% of which are strong (using the same definition of weak and strong used for the mechanistic model in the main text). For the mechanistic model, such a population synchronizes with a period of approximately 24 hours, has a mean per mRNA amplitude of 2.5 a.u. during the first day and one of 4.85 a.u. once the population has synchronized. The full model is synchronized by hour 36, so our cost function favors populations that synchronize quickly. An ideal cost would be 0 and is computed according to

$$cost = 3c_{\text{sync}} + c_{\text{amp day 1}} + c_{\text{amp after sync}}$$

where the synchronization cost c_{sync} depends on the time it takes to synchronize (reach SI=0.7), the period-to-period variability of each cell (it should be small), the distribution of periods across the population (it should be tighter than the distribution of free-running periods), the difference between the synchronized population's period and 24 (it should be small), and the number of cells whose phase is near zero (this should be small, because we don't want any dead cells). The synchronization cost is computed according to

$$c_{\text{sync}} = \frac{t_{sync}}{t_{end}} + p2p + 2\max(stdSP - 0.8stdFRP, 0) + \frac{|SP - 24|}{2} + 2numFlatCells,$$

where t_{sync} is the time at which SI first reaches 0.7, p2p is the mean period-to-period variability of the mean phase trajectory, $stdSP$ is the standard deviation of the period across cells (computed once the population has synchronized), $stdFRP$ is the standard deviation of the intrinsic periods, SP is the period of the synchronized population (computed as the period of the mean phase trajectory), and numFlatCells is the number of cells whose mean phase velocity is less than 10^{-5}. If the synchronization index never reaches 0.7, then the cost is infinite.

The amplitude is penalized only if it is more than 0.3 a.u. different from the desired amplitude. The costs are computed using the mean across cells:

$$c_{\text{amp day 1}} = \max(|mean\_d1\_amp - 2.5| - 0.3, 0)$$

and

$$c_{\text{amp after sync}} = \max(|mean\_synced\_amp - 4.85| - 0.3, 0)$$

where $mean\_d1\_amp$ is computed using the peak-to-trough amplitude of the per mRNA trace (computed using a sine function, as above) for each cell during day 1 and $mean\_synced\_amp$ is computed using the mean peak-to-trough per mRNA amplitude of each cell during the days it is synchronized (typically starting mid-day 1 and going until the simulation ends after day 6).

Results

Using parameters from an optimization (λ=0.3918/h, κ=4 a.u./h, K=12 a.u., δ=-3.5 h, β= 0.2137 1/a.u.), we simulate the TTX and wash using different population demographics. First, we vary the percentage of sustained oscillators (Figure S16). Second, we vary the type of cell at each network hub (cells with the greatest number of outputs are called hubs) (see Figure S17).

Results using the per mRNA reporter

To demonstrate the similarity between the full and reduced models, we use the same measures on both models. The synchronization index (SI) is computed using the Wavos wavelet analysis on the trace of per mRNA from each cell.

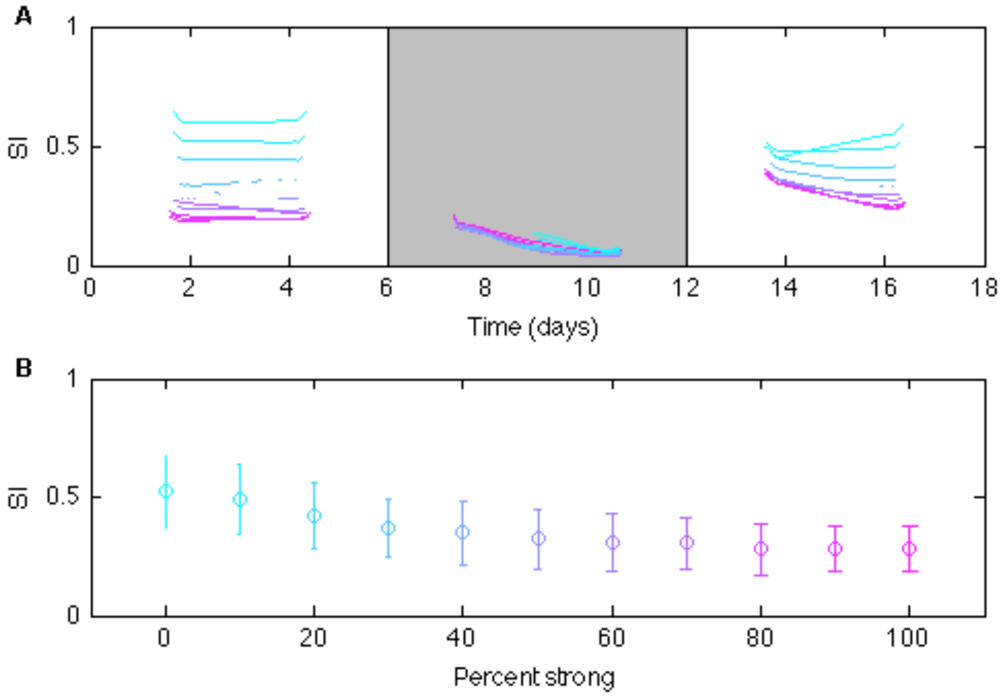

Figure S16. Networks with more weak oscillators synchronize better. We measure the synchronization index (SI) of simulated per mRNA concentrations in the coupled (days 1-6), TTX-uncoupled (days 7 - 12), and recovery (days 13-18) conditions and compare the results across different population configurations. We show the effects of varying the percentage of strong oscillators. For each configuration-type, we run 56 simulations. (A) The mean SI is shown over time for populations of 0% (cyan), 10%, 20%, 30%, 40%, 50%, 60%, 70%, 80%, 90%, and 100% (magenta) strong cells. For each simulation, we compute the mean SI for days 3 to 6 after the wash. (B) We show the mean and standard deviation across simulations using the same color scheme.

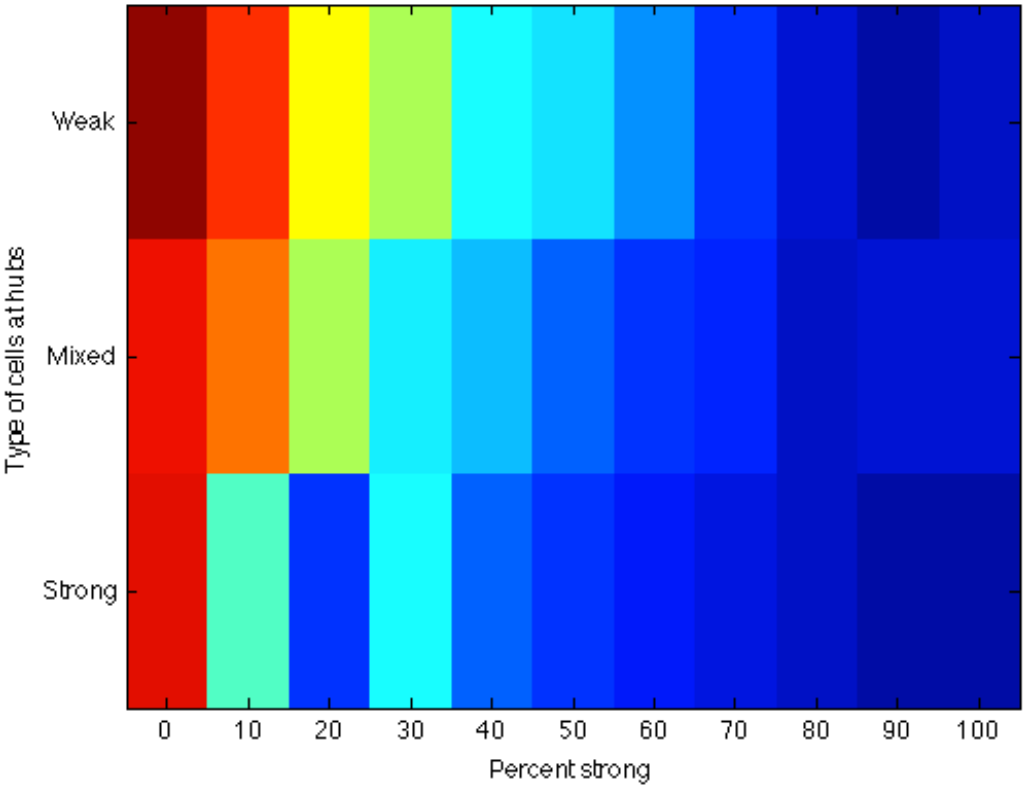

Figure S17. Networks with weak oscillators at hubs synchronize better. We measure the synchronization index (SI) of simulated per mRNA concentrations in the recovery conditions and compare the results across different network configurations. We show the effects of varying the phenotypes of cells at network hubs, in addition to varying the percentage of strong oscillators. For each configuration-type, we run 56 simulations. For each simulation, we compute the mean SI for days 3 to 6 after the washout. We show the mean SI, color-coded according to the rainbow (red indicates SI = 1, blue indicates SI = 0).

Results using the phase variable directly

The ultimate goal of this modeling project is to understand the process of re-synchronization. For this reduced model, we have a variable tracking phase. By computing the SI directly from the phase variable, we access details missing from the full model's phase estimation. In Figures S18 and S19, we show results from the same simulations shown in Figures S16 and S17. Here, we use the phase variable. When the SI is averaged over time, it is clear that the two SI methods are in agreement – the average quality of synchronization is higher when damped cells dominate (whether it is by number or by position). However, there are oscillations in this direct SI that are not present in the WAVOS-estimated SI. The oscillations reflect the fact that cells are being pulled into and out of sync throughout their cycles. The phase variable is tracking even minute changes in the phase that are not readily apparent from the per mRNA trace computed using that variable. Additionally, wavelet analysis smooths the per mRNA trace, leading to a phase estimation with less high frequency variation over time. Thus, the oscillations represent phase adjustments that are present, but that are not detectable by a reporter. The important information is a smoother line (such as the mean value) throughout the cycle.

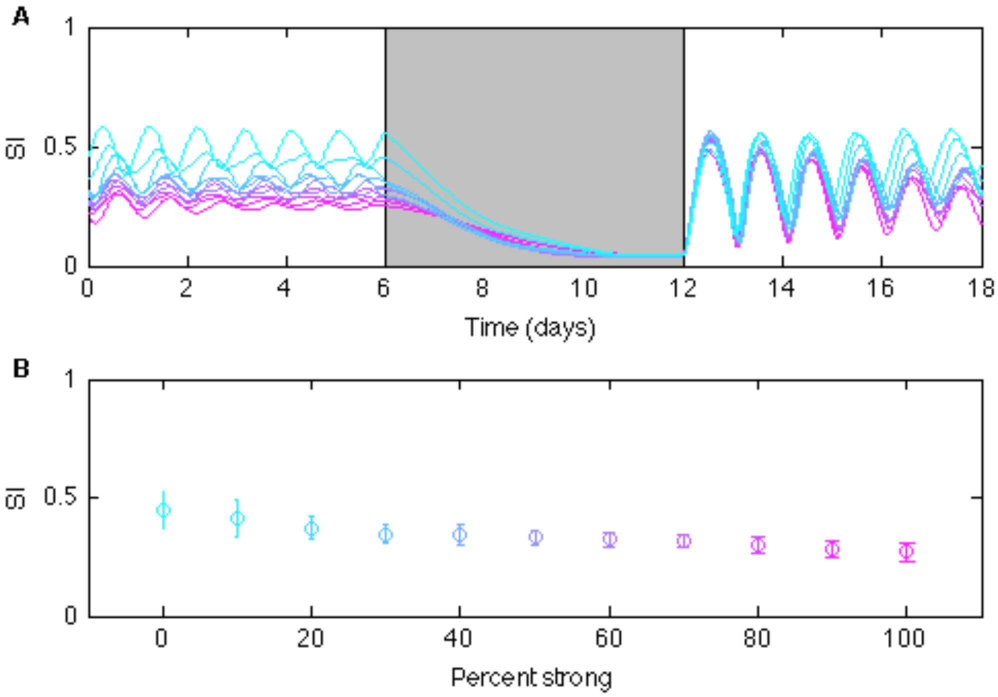

Figure S18. Networks with more weak oscillators synchronize better. We measure the synchronization index (SI) using the phase variable of a simulated population in the coupled (days 1-6), TTX-uncoupled (days 7 - 12), and recovery (days 13-18) conditions and compare the results across different population configurations. We show the effects of varying the percentage of strong oscillators. For each configuration-type, we run 56 simulations. (A) The mean SI is shown over time for populations of 0% (cyan), 10%, 20%, 30%, 40%, 50%, 60%, 70%, 80%, 90%, and 100% (magenta) strong cells. For each simulation, we compute the mean SI for days 3 to 6 after the wash. (B) We show the mean and standard deviation across simulations using the same color scheme.

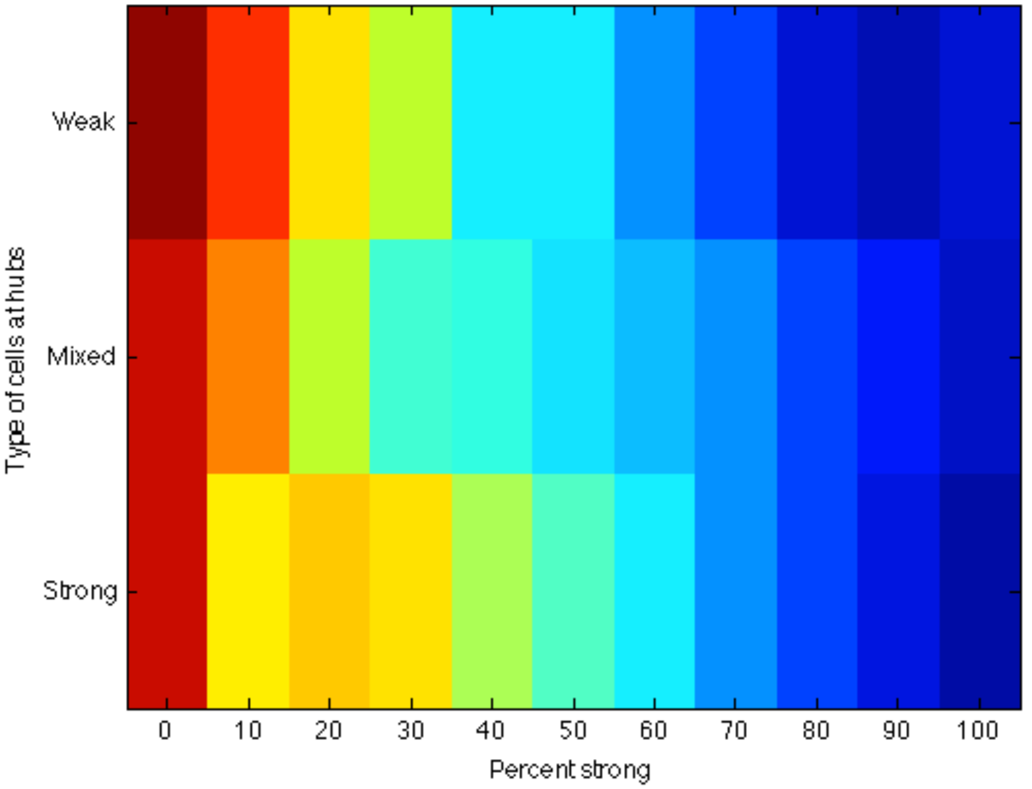

Figure S19. Networks with weak oscillators at hubs synchronize better for most population demographics. We measure the synchronization index (SI) of simulated per mRNA concentrations in the recovery conditions and compare the results across different network configurations. We show the effects of varying the phenotypes of cells at network hubs, in addition to varying the percentage of strong oscillators. For each configuration-type, we run 56 simulations. For each simulation, we compute the mean SI for days 3 to 6 after the washout. We show the mean SI, color-coded according to the rainbow (red indicates SI = 1, blue indicates SI = 0).

References

Granada AE, Herzel P (2009) How to achieve fast entrainment? The timescale to synchronization, PLoS ONE, 4(9): e7057.

Hafner M, Koeppl H, Gonze D (2012) Effect of network architecture on synchronization and entrainment properties of the circadian oscillations in the suprachiasmatic nucleus. PLoS Computational Biology 8: e1002419.

Kuramoto Y (1984), *Chemical oscillations,waves, and turbulence*. Springer-Verlag, Berlin.

Leloup JC, Goldbeter A (2003) Toward a detailed computational model for the mammalian circadian clock. PNAS 100: 7051-7056.

To TL, Henson MA, Herzog ED, Doyle III FJ (2007) A Molecular Model for Intercellular Synchronization in the Mammalian Circadian Clock. Biophys J 92: 3792-3803.

Vasalou C, Herzog ED, Henson MA (2009) Small-World Network Models of Intercellular Coupling Predict Enhanced Synchronization in the Suprachiasmatic Nucleus. J Biol Rhythms 24: 243-254.
